# Systemic and local decorin levels mirror the clinical course of pancreatic cancer

**DOI:** 10.1002/2056-4538.70066

**Published:** 2025-12-14

**Authors:** Maja Svensson, Sophie Lehn, Hedda Jacobsen, Sofie Olsson Hau, Göran Jönsson, Kristian Pietras, Karin Jirström

**Affiliations:** ^1^ Division of Oncology and Therapeutic Pathology, Department of Clinical Sciences Lund University Lund Sweden; ^2^ Division of Translational Cancer Research, Department of Laboratory Medicine Lund University Lund Sweden; ^3^ Department of Palliative Care and Advanced Home Health Care Primary Health Care Skåne Malmö Sweden; ^4^ Division of Oncology, Department of Clinical Sciences Lund University Lund Sweden; ^5^ Department of Clinical Genetics, Pathology and Molecular Diagnostics Skåne University Hospital Lund Sweden

**Keywords:** decorin, serum, pancreatic cancer, B cells, T cells, macrophages, chemotherapy prognosis

## Abstract

Despite significant progress in oncology research, pancreatic cancer remains inherently difficult to treat, and the mechanisms underlying therapeutic resistance remain unresolved. Decorin (DCN), a member of the family of small leucine‐rich proteoglycans, has emerged as a versatile actor in various malignant diseases. The aim of this study was to further explore the potential clinical significance of DCN in pancreatic cancer, both regarding its dynamics in serum during chemotherapy and its compartmental and cellular distribution in tumour tissue. To this end, repeated on‐treatment levels of soluble DCN were measured using proximity extension assay in 124 patients enrolled in a prospective, observational clinical study, inviting patients diagnosed with pancreatic or other pancreatobiliary‐type periampullary adenocarcinoma eligible for adjuvant (*n* = 30) or first‐line palliative (*n* = 94) chemotherapy. Multiplexed immunofluorescence was applied to map DCN and the associated immune landscape in resected tumours. The results showed increasing levels of DCN in serum after initiation of chemotherapy in palliative, but not in adjuvant, patients. A higher rate of change of serum DCN was an independent adverse prognostic factor in both treatment settings. There was no significant association between systemic levels and local DCN expression. Varying expression of DCN was denoted in both tumour cells, immune cells and stroma, but the prognostic significance was mainly assigned to its expression in B cells. In particular, a higher percentage of DCN positive B cells, overall and in interaction with tumour cells, were independent predictors of shorter survival. In summary, this study is the first to demonstrate the potential clinical utility of on‐treatment monitoring of systemic DCN in patients with pancreatic cancer. The findings also provide interesting leads for further research into how DCN may interact with the immune microenvironment to promote tumour development and the emergence of chemoresistance.

## Introduction

Research into new methods to treat pancreatic ductal adenocarcinoma (PDAC), one of the most lethal malignant neoplasms worldwide, is still on a long uphill journey. Surgical resection, the only possibility of attaining cure, is achievable in merely a fifth of patients. Five‐year survival rates after curative surgery in patients with histologically confirmed PDAC are approximately 20%, and long‐term survival is mainly seen in cases with favourable histopathological factors [1]. The situation is further aggravated by the fact that almost all PDAC tumours are inherently resistant to new types of oncological treatment, leaving conventional chemotherapy the only option for systemic treatment in both the adjuvant and palliative setting, where the latter applies to the vast majority of the patients.

In order to find new treatment strategies to improve the outlook for patients with PDAC, new therapeutic targets need to be identified. In addition, there is also an evident need for an increased understanding of how various factors in the tumour microenvironment as well as in the systemic host response contribute to the highly malignant tumour phenotype, and to the emergence of chemoresistance, which is often seen within a matter of weeks.

In a recent study investigating associations between immune‐related serum markers and quality of life in the first 100 patients enrolled in the CHAMP study, a prospective, observational study of patients with PDAC and other periampullary cancers [2], decorin (DCN) was found to be among the top up‐regulated proteins after initiation of chemotherapy treatment. DCN is a member of the family of small leucine‐rich proteoglycans and the name derives from its ability to ‘decorate’ collagen in the extracellular matrix [3]. Since its initial characterisation as a key regulator in collagen fibrillogenesis, soluble DCN has been assigned multiple functions in various biological processes such as wound repair, angiogenesis, autophagy, inflammation and immune regulation, with extensive implications for an important role in various autoimmune and inflammatory diseases [4]. In the context of malignant disease, DCN was initially proposed to be a ‘guardian from the matrix’, with far‐reaching abilities to antagonise neoplastic growth [5]. This simplified perception has since then been challenged. In PDAC, DCN has been found to be upregulated in the extracellular matrix compared to normal pancreas. It has been shown to suppress tumour growth *in vitro*, but by contrast also to attenuate the effect of gemcitabine, suggesting its involvement in chemotherapy resistance [6]. DCN has also been shown to be released by ferroptotic cancer cells in PDAC development and, when released, acts as a damage‐associated molecular pattern (DAMP) recognised by macrophages via advanced glycosylation end‐product specific receptor (AGER), triggering an inflammatory response [7].

The aim of this study was to take a holistic approach to further interrogate the potential clinical significance of DCN in patients with PDAC. To this end, repeated on‐treatment levels of soluble DCN were analysed in 124 patients enrolled in the CHAMP study, followed by mapping of DCN and the associated immune landscape in resected tumours using multiplexed immunofluorescence.

## Materials and methods

### Patient cohort

The Chemotherapy, Host response And Molecular dynamics in Periampullary adenocarcinoma (CHAMP) study (Clinicaltrials.gov identifier NCT03724994) is a prospective, observational, single‐arm, clinical study at Skåne University Hospital, Sweden, inviting patients diagnosed with pancreatic or other periampullary adenocarcinoma eligible for adjuvant, neoadjuvant or first‐line palliative chemotherapy [8]. Ethical approval for the CHAMP study has been obtained from the Regional Ethical Review Board (dnr LU 2018/13) and the Swedish Ethical Review Authority (amendments 2021‐00166 and 2021‐06065). Before entry, the patients received information about the study from their oncologist or research nurse and provided written consent if they wanted to participate. The study protocol follows the agreements of the Declaration of Helsinki. Enrolment was closed on 31 December 2024, and this study encompasses all 124 patients included up until 31 December 2022, 30 of whom were treated with adjuvant intent and 94 with palliative intent. The patients were treated according to standard of care and study blood samples were collected before each new treatment cycle. Clinical information was collected from patient records, and all resected specimens and biopsies have been histopathologically re‐evaluated by a senior pathologist (KJ). Individual tissue microarrays (TMAs) were constructed as ‘single‐patient tissue chips’ (SPTCs), containing multiple 1 mm cores representing all formalin‐fixed paraffin‐embedded (FFPE) donor blocks from each resected primary tumour, as previously described in detail [8]. For this study, SPTCs were constructed from 27/30 patients, excluding three patients who underwent surgery in other hospitals in Sweden (*n* = 2) or Germany (*n* = 1). Last follow‐up was on 31 May 2025. Overall survival (OS) was calculated from the date of diagnosis to death or last follow‐up. Recurrence free survival (RFS) in resected cases was calculated from the date of surgery to the last follow‐up. A flow chart showing the number of patients included in each stage of the analyses is provided in supplementary material, File S1.

### Serum samples and proximity extension assay

From all 124 patients in the CHAMP study, serum samples from baseline (*n* = 121) 1 month (*n* = 108), 3 months (*n* = 87), and 6 months (*n* = 52) of treatment, based on availability, were selected for proximity extension assay (PEA) using a panel of 92 immuno‐oncology related proteins [9, 10], one of which is DCN, and reported as normalised protein expression (NPX) on log_2_ scale. In brief, upon collection, blood samples were centrifuged, and serum was collected and cryopreserved in −80°C until analysis. Due to the different objectives, the sample selection, outlined in supplementary material, File S2, differed from the previous study [2], which included 75 of the patients analysed herein.

### Multiplex immunofluorescence

The expression of DCN was evaluated alongside tumour and immune cells on SPTCs from 27 patients using a 6‐plex immunofluorescent panel consisting of anti‐DCN, anti‐CD4, anti‐CD8, anti‐CD20, anti‐CD68, and anti‐pan‐cytokeratin (PanCK). A detailed description of the staining protocol, the antibody‐fluorophore pairings, incubation times and dilutions are given in supplementary material, File S3. Multispectral images of each SPTC were acquired in the PhenoImager HT 2.0 system (Akoya Biosciences, Marlborough, MA, USA) at 20× magnification, including spectral unmixing in InForm (version 3.0, Akoya Biosciences).

### Image analysis

After image acquisition, downstream analysis was performed in QuPath version 0.6.0‐rc2 [11]. Only TMA cores of sufficient quality and containing cancer cells were included in the downstream analysis. A detailed description of the image analysis workflow is found in supplementary material, File S3. In brief, cell segmentation was performed using the InstanSeg [12] extension in QuPath, and positive cell classifiers were trained for each marker. A tissue classifier was trained to identify tumour (PanCK^+^), stromal and immune rich areas.

The SPTC of one patient consisted of samples from two specimens, one from a Whipple's procedure and another from a left‐sided pancreatectomy 1 year later, and was treated as two cases.

### Statistical analyses

Statistical analyses were performed using the programming language R version 4.2.2 and R Studio version 2024.04.2. To categorise patients into two groups based on a continuous variable and survival outcome, the function *surv_cutpoint*() was used, applying maximally selected rank statistics to calculate the optimal prognostic cut‐off. In the Kaplan–Meier analyses, log rank tests were used to calculate *p* values. A Cox proportional hazards regression model was applied, using the *coxph*() formula from the *survival* R package (version 3.5‐5), to fit a regression model and generate hazard ratios (HR) and 95% confidence intervals (CI). Comparisons of serum DCN levels between timepoints were made using Wilcoxon signed‐rank test and Spearman's correlation coefficients were calculated using the function *cor.test*(), both from the R package *stats* (version 4.2.2).

## Results

### Patient demographics and clinicopathological characteristics

Patient demographics and clinicopathological characteristics for patients treated in adjuvant (*n* = 30) and palliative (*n* = 94) settings in the study cohort are shown in Table 1. The vast majority of the patients had tumours originating in the pancreas, and all tumours originating in the Ampulla of Vater or distal bile duct were of pancreatobiliary type. Median overall survival (OS) was 38.6 months (range 3.6–76.4) in adjuvant patients and 8.4 (range = 1.0–75.4) months in palliative patients. Six of the operated patients received neoadjuvant chemotherapy for downstaging purposes, and two within the randomised trial NORPACT [13], one of whom did not receive adjuvant chemotherapy.

**Table 1:** Patient demographics and clinicopathological characteristics in the CHAMP cohort

	Adjuvant, *n* = 30	Palliative, *n* = 94
Sex		
Male	21 (70%)	42 (44.7%)
Female	9 (30%)	52 (55.3%)
Age		
Median (range)	69 (45.9–79.8)	70.2 (38.4–83.3)
Tumour origin		
Pancreas	28 (93.3%)	89 (94.7%)
Distal bile duct	1 (3.3%)	4 (4.3%)
Ampulla of Vater	1 (3.3%)	1 (1.1%)
Performance status at baseline		
0	17 (56.7%)	16 (17%)
1	8 (26.7%)	50 (53.2%)
2	5 (16.7%)	22 (23.4%)
3	0 (0%)	6 (6.4%)
Regimen		
FOLFIRINOX*	9 (30%)	40 (42.6%)
Gemcitabine + Nab‐paclitaxel	2 (6.7%)	37 (39.4%)
Gemcitabine	10 (33.3%)	17 (18.1%)
GemCap	9 (30%)	0 (0%)
Metastatic		
No	–	34 (36.2%)
Yes	–	60 (63.8%)
Surgery		
Whipple	18 (60%)	–
Distal pancreatectomy	9 (30%)	–
Total pancreatectomy	3 (10%)	–
Tumour grade		
G1	3 (10%)	–
G2	11 (36.7%)	–
G3	5 (16.7%)	–
NA	11 (36.7%)	–
T stage		
T1	4 (13.3%)	–
T2	20 (66.7%)	–
T3	6 (20%)	–
R status		
R0	13 (43.3%)	–
R1	17 (56.7%)	–
N stage		
N0	8 (26.7%)	–
N1	11 (36.7%)	–
N2	10 (33.3%)	–
NA	1 (3.3%)	–
Vascular invasion		
No	16 (53.3%)	–
Yes	14 (46.7%)	–
Lymphatic invasion		
No	15 (50%)	–
Yes	15 (50%)	–
Perineural invasion		
No	10 (33.3%)	–
Yes	20 (66.7%)	–

* Fluorouracil, irinotecan, oxaliplatin, leucovorin. R status: R0 = no involved margins; R1 = involved margins. N status: N0 = no metastatic lymph nodes; N1 = 1–3 metastatic lymph nodes; N2 ≥ 4 metastatic lymph nodes.

### Trajectories of DCN levels in serum during adjuvant and first‐line chemotherapy treatment

On‐treatment blood sampling was performed from before the start of treatment, referred to as the baseline (BL), until the end of treatment or progression. A median of three serum samples per patient (range 1–7) were analysed and three patients had a missing BL sample. Five of the adjuvant patients were excluded from the survival analysis as blood sampling was only performed during neoadjuvant treatment. One palliative and one adjuvant patient with Lynch syndrome, who received immunotherapy after first‐line and adjuvant chemotherapy, respectively, were excluded from the survival analyses. Another patient, treated with first‐line palliative chemotherapy who was eventually diagnosed with a *BRCA2*‐mutated tumour and switched to PARP‐inhibitor, was also excluded from the survival analyses. A flow chart of the number of patients included in different stages of the analyses is included in supplementary material, File S1.

**Figure 1:**
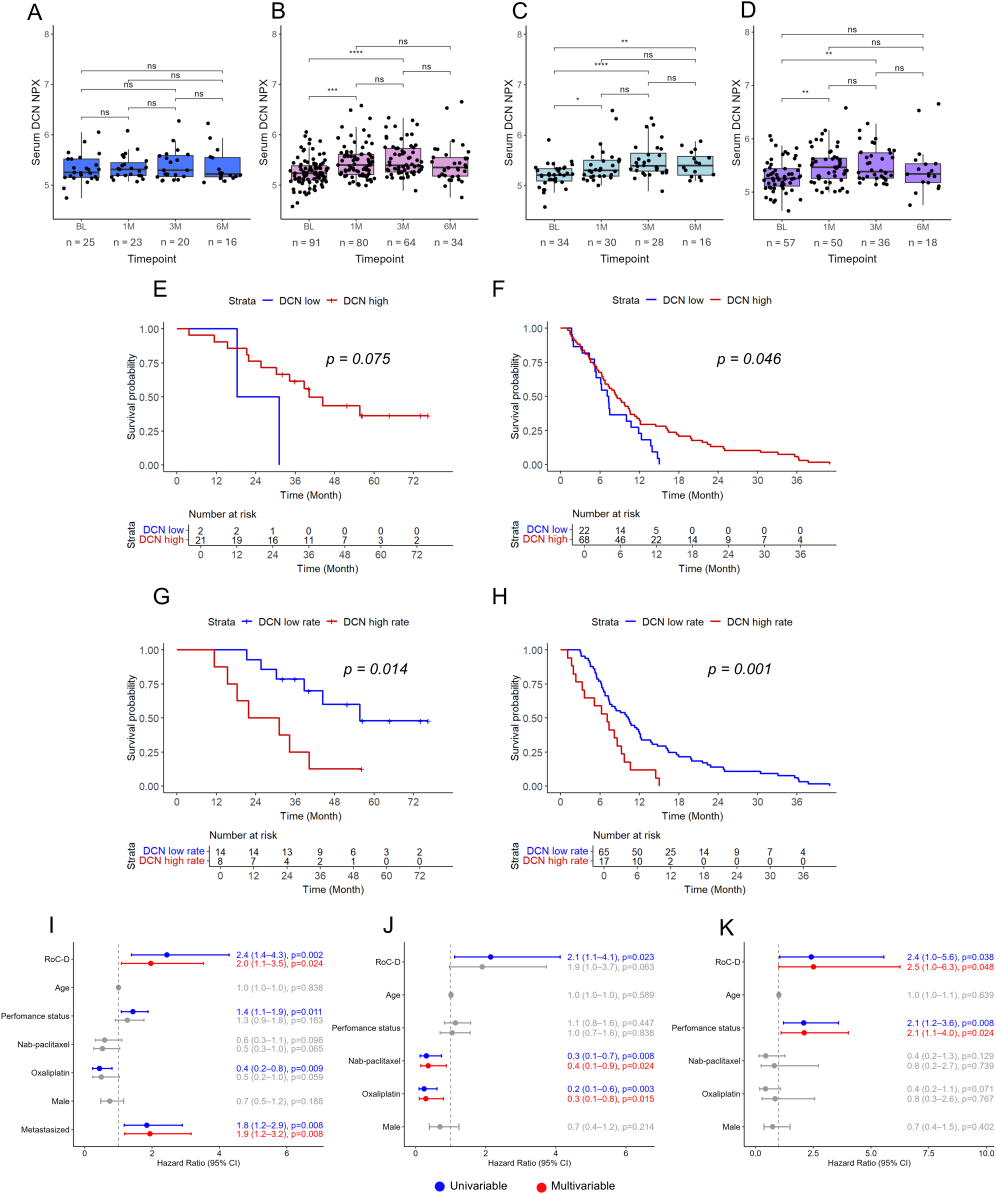
Levels and prognostic value of DCN in serum. (A–D) boxplots of levels of DCN in serum at different timepoints during chemotherapy in (A) adjuvant and (B) palliative patients, and palliative patients stratified into (C) locally advanced and (D) metastatic disease. The Wilcoxon signed‐rank test was used to calculate the difference between the timepoints. ns, not significant; *p < 0.05; **p < 0.01; ***p < 0.001; ****p < 0.0001. NPX, normalised protein expression; 1M, 1 month; 3M, 3 months; 6M, 6 months; BL, baseline. Kaplan–Meier analysis of levels of DCN at baseline, dichotomised into high and low according to the optimal prognostic cut‐off, and overall survival in (E) adjuvant (cut‐off = 4.94 NPX) and (F) palliative patients (cut‐off = 5.09 NPX). Kaplan–Meier analysis of the rate of change of serum DCN, dichotomised into high and low according to the optimal prognostic cut‐off, and overall survival in (G) adjuvant (cut‐off = 0.15 NPX/month) and (H) palliative patients (cut‐off = 0.38 NPX/month). The *p* values in the Kaplan–Meier analyses were calculated using the log‐rank test of comparisons of high and low levels, or rates of change, of DCN. Forest plots showing univariable and multivariable hazard ratios (HR) for death with 95% confidence intervals (CI) according to the dichotomised rate of change of serum DCN (RoC‐D) and clinicopathological factors in (I) all palliative patients, and palliative patients stratified into (J) metastatic and (K) locally advanced disease, respectively.

As shown in Figure 1A, B, levels of serum DCN increased from BL to 1 month (1M), 3 months (3M), and 6 months (6M), respectively, in palliative patients, but not in adjuvant patients. Of note, 1M, 3M, and 6M blood samples were collected at intervals of 0.45–1.55, 2–4, and 4.5–7.5 months from BL, respectively. The median BL levels were 5.25 NPX for adjuvant patients and 5.23 NPX for palliative patients. As further shown in Figure 1C, D, DCN levels increased from BL to all later timepoints in palliative patients with locally advanced disease, and from BL to 1M and 3M in patients with metastatic disease.

### The rate of change rather than absolute DCN levels in serum is prognostic in both the adjuvant and palliative setting

An optimal prognostic cut‐off for serum DCN at different timepoints during treatment was determined separately for adjuvant and palliative patients. Kaplan–Meier analysis showed that no association between DCN levels at BL and OS was found in adjuvant patients (Figure 1E; *p* = 0.075), whereas in palliative patients, high DCN levels at BL were associated with a longer OS (Figure 1F; *p* = 0.046). As further shown in supplementary material, File S4, high DCN levels at 3M were also associated with a shorter OS in adjuvant patients, whereas it was not prognostic at any other timepoint in palliative patients. DCN levels were significantly associated with longer RFS in adjuvant patients at 3M of treatment but not at any other timepoint (*p* = 0.033; supplementary material, File S5).

Next, the rate of change in serum DCN levels (RoC‐D) following chemotherapy initiation was calculated in patients who had a BL sample and a second sample (*n* = 104), as the difference in NPX between the second and BL measurements divided by the time elapsed between these timepoints. The median time between BL and the second sample was 0.9 months (range 0.5–2.3). Kaplan–Meier analysis showed that a higher RoC‐D was significantly associated with shorter survival in both adjuvant (Figure 1G; *p* = 0.014) and palliative (Figure 1H; *p* = 0.001) patients. The prognostic value of the RoC‐D in adjuvant patients was confirmed in univariable Cox regression analysis and remained prognostic for OS and RFS in multivariable analysis (supplementary material, File S6). In palliative patients, the prognostic value of the RoC‐D was confirmed in univariable analysis and remained prognostic in multivariable analysis adjusted for performance status, chemotherapy regimen and metastatic stage (Figure 1I). When further dividing palliative patients into those having locally advanced and metastatic disease, RoC‐D remained an independent prognostic factor only in the former (Figure 1J) but not in the latter (Figure 1K). To further validate these findings, we examined the prognostic significance of the RoC‐D stratified into tertiles (supplementary material, File S7), whereby a significant difference in OS was seen between the first and third tertiles (*p* = 0.021) in palliative patients. Moreover, multivariable Cox regression using continuous values of the RoC‐D confirmed the independent prognostic value in palliative patients. DCN was not significant in any of these analyses in adjuvant patients.

### Distribution and prognostic value of DCN in tumour cells, immune cells and stroma

**Figure 2:**
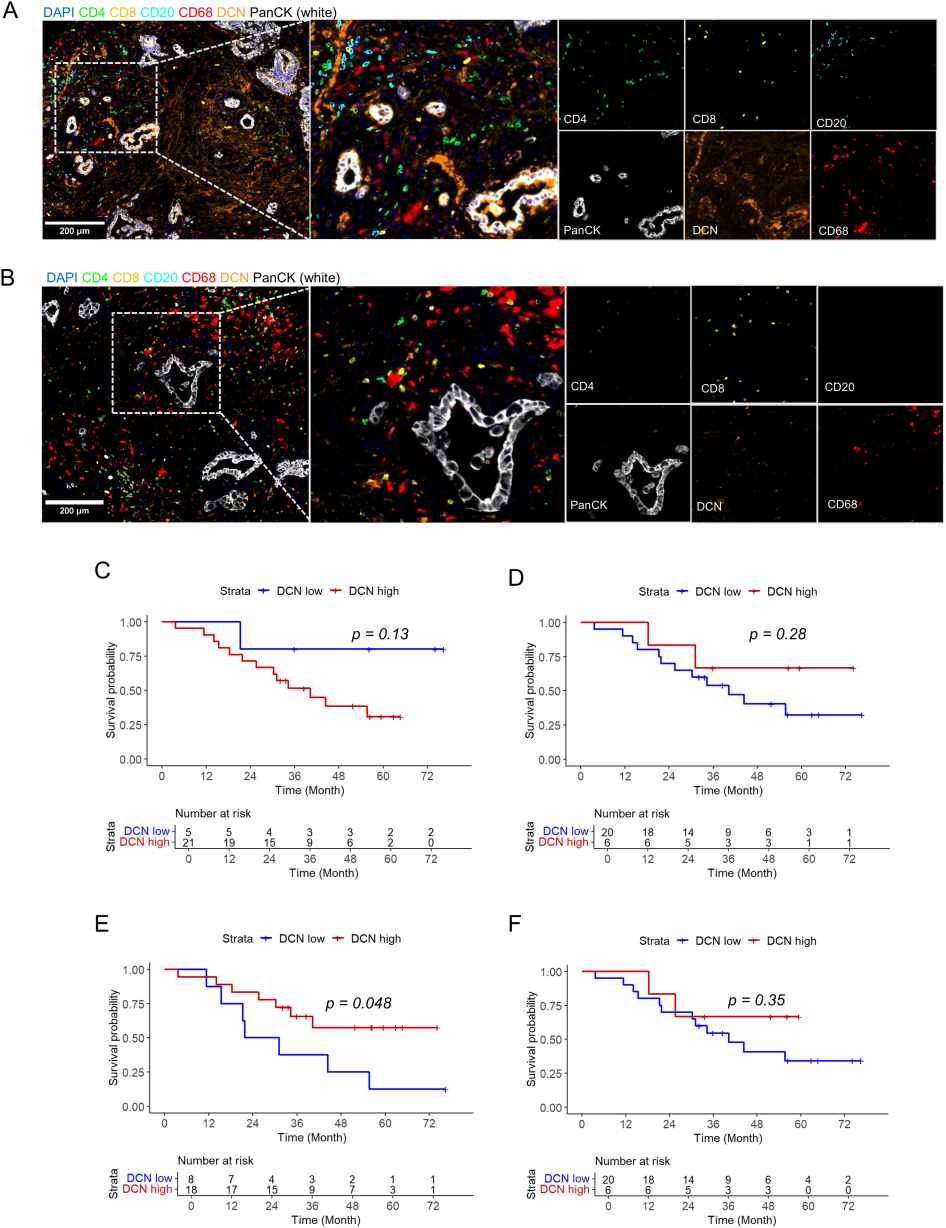
Prognostic value of compartment‐specific DCN expression. Multiplex immunofluorescent images of two tissue cores stained for CD4 (CD4^+^ T cells, green), CD8 (CD8^+^ T cells, yellow), CD20 (CD20^+^ B cells, cyan), CD68 (CD68^+^ macrophages, red), DCN (orange) and PanCK (tumour cells, white), representing (A) one case with a high fraction of DCN in the tumour cells, and (B) one case with a low fraction of DCN in the tumour cells. Kaplan–Meier analysis of overall survival in relation to (C) the fraction (cut‐off 12% DCN^+^ PanCK^+^ cells/total PanCK^+^ cells) and (D) intensity of DCN in tumour cells [cut‐off 8.04 arbitrary units (AU)], (E) DCN intensity in stroma (cut‐off 32.39 AU) and in (F) immune‐rich areas (cut‐off 35.47 AU). The *p* values were calculated using the log‐rank test of comparisons of high and low DCN expression.

In order to assess the expression of DCN in tumour cells, stroma and immune cells, SPTCs from 27 patients were stained with a 6‐plex immunofluorescent panel including DCN, CD4, CD8, CD20, CD68, and PanCK. The panel allowed for identification of DCN, CD4^+^ T cells, CD8^+^ T cells, CD20^+^ B cells, CD68^+^ macrophages, and PanCK^+^ tumour cells. Representative images of the staining from two cases are shown in Figure 2A, B. Information on the total number of donor blocks, TMA cores, overall and used in the analyses, is provided in supplementary material, File S8.

First, we mapped the compartment‐specific distribution of DCN expression and, as shown in supplementary material, File S9, DCN expression in tumour cells, stroma and immune rich compartments did not differ between treatment naïve and treated tumours.

Kaplan–Meier analyses showed that neither the fraction nor the intensity of DCN in tumour cells was prognostic (Figure 2C, D), that a high intensity of DCN in the stroma was associated with a longer OS (Figure 2E; *p* = 0.048), and that the intensity of DCN in immune‐rich areas was not prognostic (Figure 2F). Stromal DCN expression did not remain significant when adjusting for N stage in multivariable Cox regression analysis (supplementary material, File S10). As shown in supplementary material, File S11, N stage was the only prognostic conventional clinicopathological parameter for OS, and performance status and N stage for RFS.

### DCN expression is enriched in B cells and a higher proportion of DCN positive B cells is associated with shorter survival

**Figure 3:**
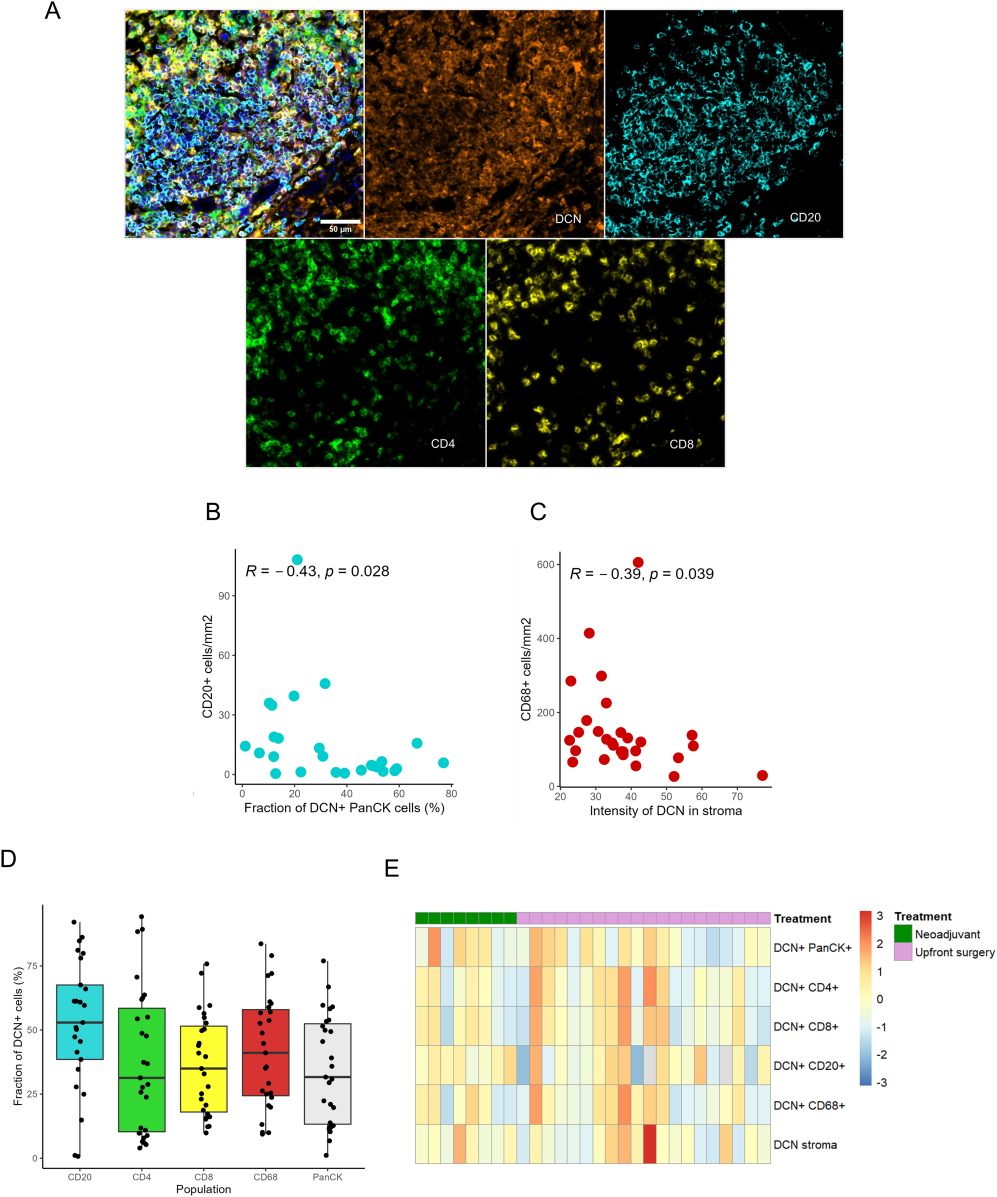
DCN expression in different immune cell populations. (A) Representative multiplex immunofluorescence image of DCN positive immune cells. Spearman's correlation analyses between (B) the number of CD20^+^ B cells and the fraction of DCN in PanCK^+^ tumour cells, and between (C) the number of CD68^+^ macrophages and the intensity of DCN in stroma. (D) Fraction of DCN per cell population. (E) Heatmap of the mean fractions of DCN in cell populations per patient.

Next, we examined in more detail the expression of DCN in different immune cell subsets. A multiplexed immunofluorescence image of DCN positive immune cells is shown in Figure 3A. When investigating the relationship between the abundance of different immune cell subsets and the fraction of DCN positive tumour cells and the intensity of DCN in stroma, respectively, we found that the number of CD20^+^ B cells was significantly lower in cases with a higher fraction of DCN positive tumour cells (Figure 3B). Moreover, the number of CD68^+^ macrophages were significantly lower in cases with high DCN expression in stroma (Figure 3C). The abundance of CD4^+^ T cells, CD8^+^ T cells did not differ according to DCN expression in either tumour cells or stroma (supplementary material, File S12).

We further analysed the proportion of DCN positive CD4^+^ T cells, CD8^+^ T cells, CD20^+^ B cells and CD68^+^ macrophages, respectively, whereby the highest proportion was seen in CD20^+^ B cells (Figure 3D). An overview of the interpatient distribution of DCN positivity in tumour cells, in immune cell subsets and in the stromal compartment unveiled some, but no tangible, degree of heterogeneity (Figure 3E). DCN gene expression in immune cells was validated in public single‐cell gene expression data using three independent datasets in CZ CELLxGENE Discover [14, 15, 16, 17], and with single immunohistochemical staining in independent PDAC samples from a retrospective cohort [18], as shown in supplementary material, File S13.

**Figure 4:**
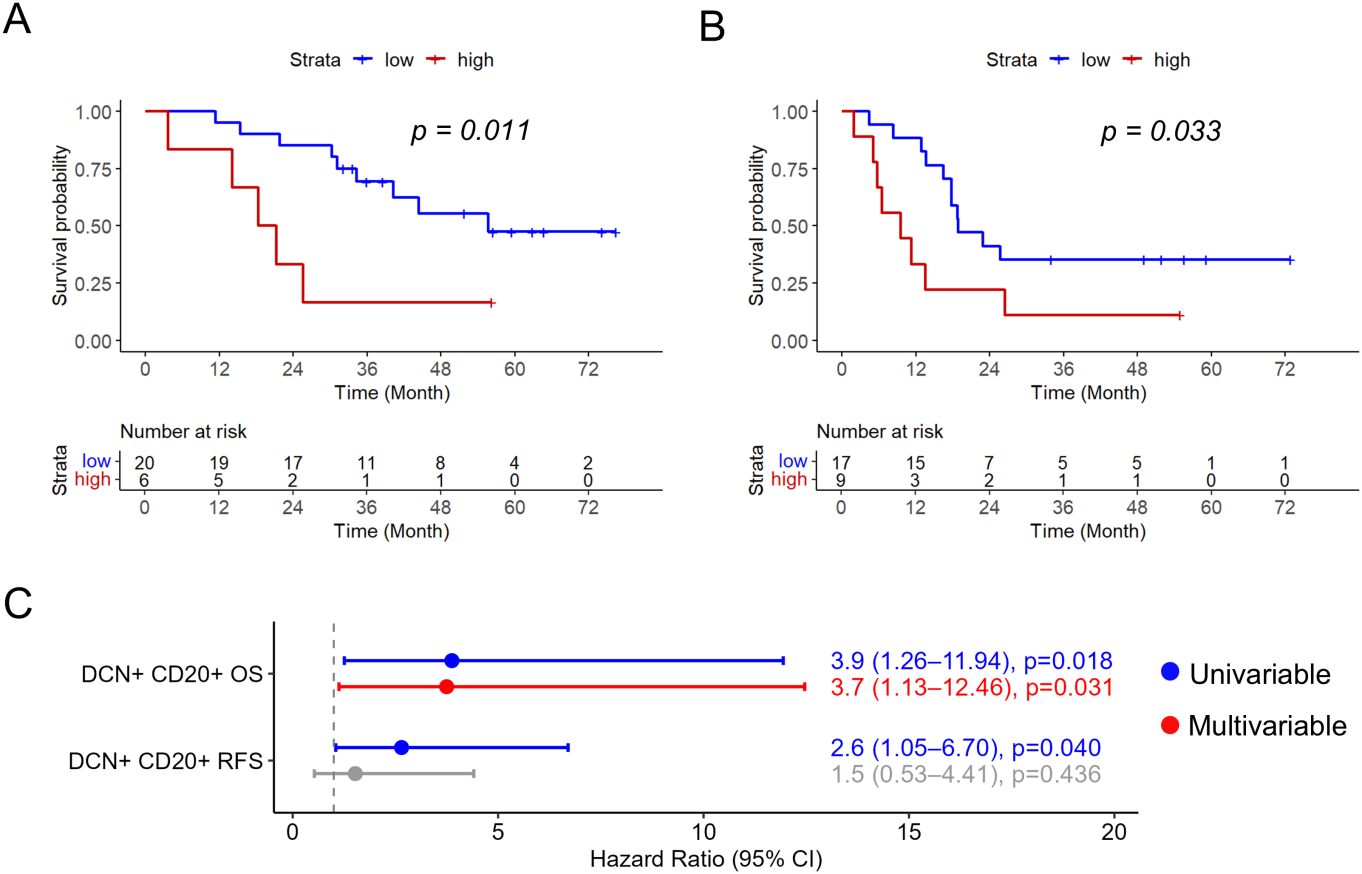
Prognostic value of DCN expression in B cells. Kaplan–Meier analysis of (A) overall survival and (B) recurrence‐free survival in relation to the fraction of DCN^+^ CD20^+^ B cells (cut‐off for OS 67.56% and cut‐off for RFS 61.23%). (C) Univariable and multivariable Cox regression analysis of hazard ratios for death and recurrence in relation to the fraction of DCN^+^ CD20^+^ B cells, using the same cut‐offs as in the Kaplan–Meier analyses.

Kaplan–Meier analysis showed that a high fraction of DCN positive CD20^+^ cells was associated with shorter OS (Figure 4A; *p* = 0.011) and RFS (Figure 4B; *p* = 0.033). The association with OS remained significant in multivariable Cox regression analysis, as shown in Figure 4C, but not when applying continuous units (supplementary material, File S14). DCN expression in CD4^+^ T cells, CD8^+^ T cells, or CD68^+^ macrophages was not associated with OS, whereas high fractions of DCN in CD8^+^ and CD68^+^ cells were associated with a longer RFS (supplementary material, File S15).

Correlations between clinicopathological parameters and compartment‐specific DCN expression are shown in supplementary material, File S16. The fraction of DCN expression in tumour cells correlated positively to perineural and lymphatic invasion, and the intensity of DCN in tumour cells correlated positively to vascular invasion, whereas DCN expression in the stromal compartment or in individual immune cells did not correlate to any clinicopathological parameter.

As shown in supplementary material, File S17, there was no significant association between serum DCN levels at BL and DCN expression in resected tumours.

### A higher number of CD20 ^+^ DCN ^+^ B cells and CD4 ^+^ DCN ^+^ T cells interacting with tumour cells is associated with shorter survival

**Figure 5:**
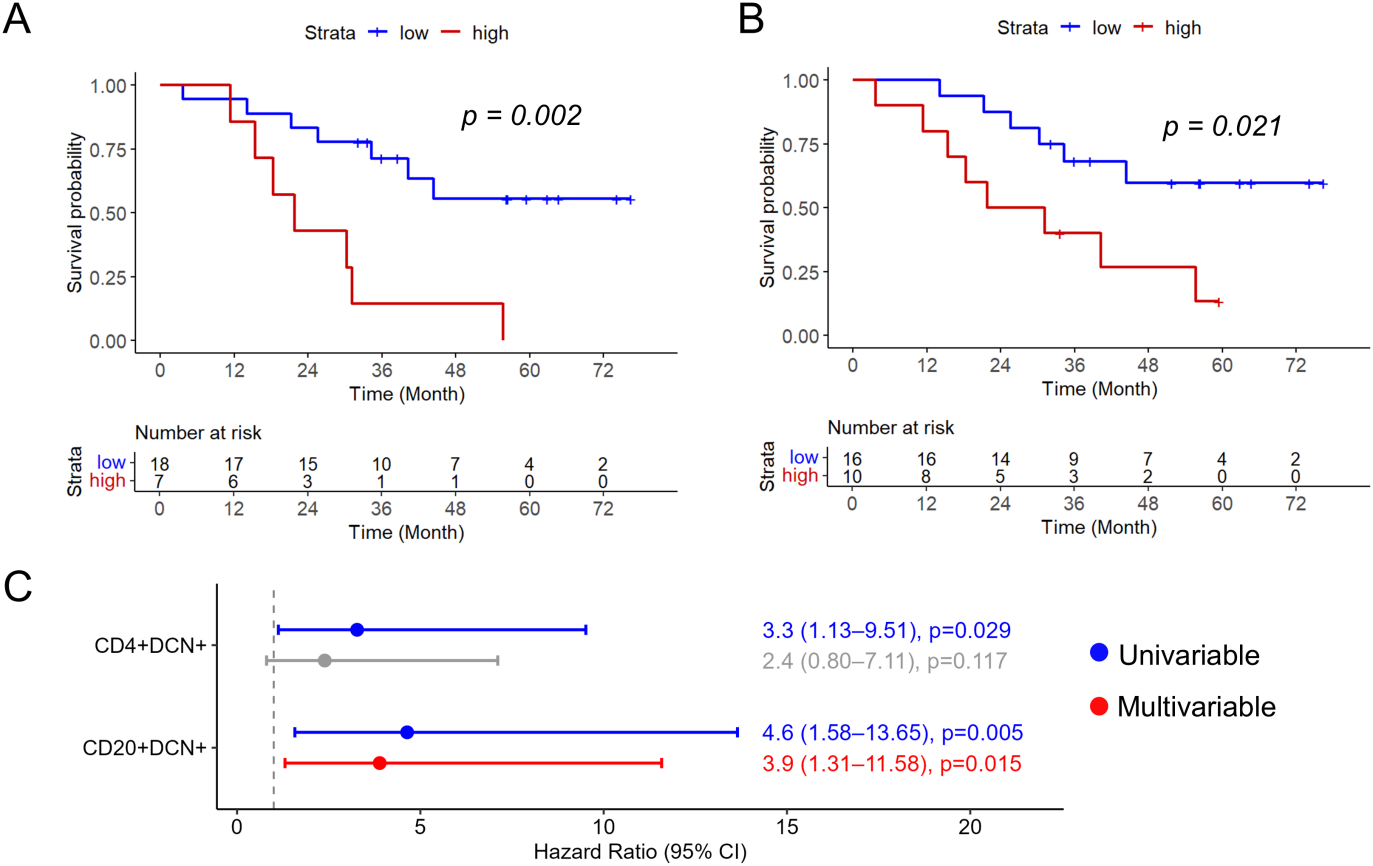
Prognostic value of the interaction of DCN‐defined CD20^+^ B cells and CD4^+^ T cells with tumour cells. Kaplan–Meier analyses of overall survival in relation to high and low fractions of (A) CD20^+^DCN^+^ B cells and (B) CD4^+^DCN^+^ T cells, interacting with tumour cells. High and low were defined according to the optimal prognostic cut‐off calculated for each immune cell population; cut‐off 2.63% for CD20^+^DCN^+^ B cells and cut‐off 2.09% for CD4^+^DCN^+^ T cells. (C) Forest plot showing univariable and multivariable Cox regression analysis of hazard ratios for death in relation to high and low fractions of CD20^+^DCN^+^ B cells and CD4^+^DCN^+^ T cells interacting with tumour cells, using the same cut‐offs as for the Kaplan–Meier analyses.

In order to investigate the spatial relationship between DCN positive (DCN^+^) immune cells and tumour cells, we calculated the number of immune cells within a distance of 15 μm of a cancer cell, as a tentative limit for cell‐to‐cell interaction [19], separately for each immune cell population and DCN status. An overview of these measurements per patient can be seen in supplementary material, File S18. After testing the hazard ratios for death and recurrence, respectively, in relation to the number of tumour‐interacting marker‐defined immune cells in univariable Cox regression analysis (supplementary material, File S19), the prognostic value of CD20^+^DCN^+^ B cells and CD4^+^DCN^+^ T cells was further investigated in Kaplan–Meier analysis applying the optimal prognostic cut‐off for each immune cell subset (Figure 5A, B). This revealed that a higher proportion of both CD20^+^DCN^+^ B cells and CD4^+^DCN^+^ T cells interacting with tumour cells was significantly associated with shorter OS. CD20^+^DCN^+^ B cells interacting with tumour cells remained prognostic in multivariable Cox regression (Figure 5C), also in adjusted analysis when applying the continuous variable (supplementary material, File S20).

## Discussion

DCN is a multifaceted protein that has been shown to participate in a variety of physiological and pathological processes, and it is becoming increasingly evident that its functions extend far beyond the extracellular matrix. This study interrogated the potential clinical significance of both systemic and tumour‐specific DCN in patients with newly diagnosed PDAC participating in a prospective, observational clinical study. We analysed the trajectories of DCN in serum during adjuvant and first‐line chemotherapy and found that increasing levels were associated with a shorter survival in both treatment settings. Moreover, while DCN levels in serum did not correlate with DCN expression in resected tumours, a comprehensive mapping of the tumour landscape unveiled variations in its compartmental and cellular distribution which could be of potential clinical importance.

This is, to our knowledge, the first study on the potential clinical significance of systemic DCN in patients with PDAC or other periampullary adenocarcinoma, and the results need to be considered from several perspectives. Firstly, significantly increasing levels from baseline, which is from initiation of chemotherapy, were only seen in palliative patients. As baseline levels did not differ between adjuvant and palliative patients, and the increase was more evident in patients with locally advanced tumours than in patients with metastatic disease, it can be assumed that this increase does not merely reflect the tumour burden, that is, that DCN in serum originates from tumour cells. While the prognostic value of baseline DCN levels was significant only in palliative patients, a higher rate of change was an independent prognostic factor in both treatment settings. Although the number of adjuvant patients was rather limited in this study, these findings should encourage validatory studies regarding the potential prognostic value of both serum DCN levels *per se* at different timepoints during treatment, but also the rate of change. The observations in this study also further underline the general value of on‐treatment disease monitoring, in particular in hard‐to‐treat cancers such as PDAC.

Indeed, studies on the potential prognostic value of systemic DCN in other cancers are scarce, and no longitudinal monitoring in this regard has been described. In a study on breast cancer, plasma DCN was reported to be increased in more advanced stages, and also to be inversely associated with stromal DCN, but no survival analyses were performed [20]. Another study reported plasma DCN levels to be lower in patients with oesophageal squamous cell carcinoma than in controls [21], and similar findings were seen in a smaller study on non‐small cell lung cancer [22].

Systemic DCN has also been studied along with other exercise‐induced myokines as a biomarker for evaluating the beneficial effect of exercise in cancer patients. Exercise has been shown to lead to upregulated systemic DCN in breast cancer patients, but without demonstrating a direct relationship between systemic DCN levels and clinical outcome [23, 24], and in prostate cancer patients, exercise had no direct effect on systemic DCN levels [25, 26, 27].

The increased levels of DCN observed after initiation of first‐line chemotherapy should also be discussed in relation to cachexia, which is particularly frequent in palliative PDAC patients, not only as a consequence of the disease but also its treatment [28]. As DCN binds to and directly inhibits myostatin [29], a potent inhibitor of muscle growth [30], it has been suggested as a potential target for maintaining the muscle mass in cachexia [31]. Although being beyond the scope of this study, our findings should encourage further research into the more precise relationship between DCN and cachexia in patients with PDAC.

We further explored the topographical and cellular localisation of DCN in the tumour microenvironment in resected tumours, which was not related to the serum levels. This is, to our knowledge, also the first study to in more detail map the compartment‐specific and cellular distribution of DCN in any type of cancer. Most previous studies have focused on DCN expression in the stromal compartment, with the prevailing conclusion that high expression is associated with a more favourable outcome [32, 33, 34, 35]. In this study, high stromal DCN expression was associated with a prolonged OS, but not in adjusted analysis. Few studies have investigated DCN expression in PDAC, but Köninger *et al* found that DCN is upregulated in tumour stroma compared to normal tissue and that DCN exerts dual actions, both inhibiting tumour growth and attenuating the effect of carboplatin and gemcitabine on pancreatic cancer cells *in vitro* [36]. They described tumour cells as being devoid of DCN [36], which is in contrast to our study, but DCN has also been shown to be upregulated in tumour cells of more aggressive bladder cancer subtypes [37].

When mapping the expression of DCN in individual immune cell subsets, we found the highest fraction of DCN positive cells among B cells, and that a higher fraction of DCN expressing B cells was an independent predictor of shorter survival. In addition, DCN positive B cells interacting with tumour cells were also associated with shorter OS. These findings indicate that the biological role of DCN in this context may differ from previously shown tumour‐suppressive functions through interaction with several receptor tyrosine kinases, including EGFR, Met, and IGF‐IR [38, 39, 40], and by attenuating the effect of TGF‐beta [41], a potent modulator in the tumour microenvironment. Hence, rather than exerting a direct anti‐tumour effect, DCN expression in B cells might reflect an immunosuppressive, pro‐tumorigenic phenotype. This interpretation aligns with the concept that the functional consequences of DCN expression are highly context‐dependent and warrants further investigation and validation. Production of DCN in B cells has been demonstrated in the setting of early stage B cell chronic lymphocytic leukaemia [42] and further clinical and preclinical studies are needed to elucidate their functional and prognostic significance in solid tumours.

In this study, DCN expression in macrophages was not prognostic, but there was an inverse association between DCN expression in stroma and macrophage abundance. DCN released by ferroptotic cells has been described as a damage‐associated molecular pattern, capable of activating macrophages and initiating inflammatory responses [7]. However, given that PDAC is typically considered an immunologically ‘cold’ tumour, characterised by a dense stroma, immune suppression and exclusion [43], it is likely that the activating effects of DCN on macrophages are overridden in this setting. Adding to this, in metastatic gastric cancer, DCN was found to be a marker of desmoplastic cancer associated fibroblasts that drive resistance to immune checkpoint inhibition [44].

While our findings offer new insights into the clinical significance of on‐treatment monitoring with serum DCN and the context dependent prognostic value of DCN in the tumour microenvironment, several limitations must be acknowledged. Firstly, the number of resected tumours in the CHAMP cohort is too small for obtaining strong statistical power. Nevertheless, the study design enables collection of valuable real‐world data, including on‐treatment biomarker monitoring. Secondly, the tissue analyses were based on the TMA technique, which must be considered in relation to a possible heterogeneous expression of the investigated markers. However, and of note, the use of individualised TMAs, representing multiple tumour areas from all representative FFPE blocks from the resected tumours, will likely give an even better overview of the tumour landscape in terms of potential heterogeneity than whole slides from selected archival tissue blocks.

In summary, we demonstrate for the first time that on‐treatment monitoring of systemic DCN may be of potential clinical utility in patients with PDAC, both in the adjuvant and in the palliative setting. Further research is encouraged to explore whether systemic DCN mirrors the tumour burden, emerging treatment resistance, the host response to the illness, or a combination of all these factors. We have also expanded the cartography of DCN in resected tumours, highlighting its interaction with the local tumour immune microenvironment, in particular with B‐cells. These findings suggest that continued research down this avenue may expand our understanding of the exceptionally immunosuppressive conditions being a hallmark of PDAC, and potentially also provide clues to new immune‐modulating therapeutic options.

## Author contributions statement

MS designed the study, developed methodology, performed statistical analysis, constructed figures and wrote the original draft; SL, HJ and KP contributed to methodology; SOH recruited patients; GJ supervised the study; KJ supervised the study, conceived and designed the project, performed statistical analysis, acquired funding and wrote the original draft. All authors reviewed and edited the original draft and read and approved the final manuscript.

## Supporting information


**File S1.** Patient flow chart
**File S2.** Serum sample overview for 124 patients
**File S3.** Detailed methods for multiplexed immunofluorescent images
**File S4.** Kaplan–Meier analyses of overall survival in relation to serum DCN levels at different timepoints in palliative and adjuvant patients
**File S5.** Kaplan–Meier analysis of recurrence free survival in relation to serum DCN levels at baseline in adjuvant patients
**File S6.** Univariable and multivariable Cox regression analyses of death and recurrence in relation to RoC‐D in adjuvant patients
**File S7.** Prognostic value of tertiles of RoC‐D in adjuvant and palliative patients
**File S8.** Number of TMA cores and donor blocks, overall and used in analysis
**File S9.** Compartment‐specific DCN expression according to neoadjuvant treatment
**File S10.** Univariable and multivariable Cox regression analyses of hazard ratios for death and recurrence in relation to DCN expression in the stromal compartment
**File S11.** Prognostic value of conventional clinicopathological parameters in adjuvant patients
**File S12.** Correlations between immune cells and DCN expression in tumour cells and stroma
**File S13.** DCN gene expression in immune cells and immunohistochemical DCN staining in a retrospective cohort
**File S14.** Univariable and multivariable Cox regression analysis of hazard ratios for death and recurrence in relation to DCN positive B cells
**File S15.** Kaplan–Meier analyses of overall and recurrence free survival in relation to different DCN positive immune cell populations
**File S16.** Correlations between DCN expression in different compartments and clinicopathological parameters
**File S17.** Correlations between and DCN expression in tumours and serum DCN levels at baseline
**File S18.** Heatmap of the number of DCN‐defined immune cells interacting with tumour cells
**File S19.** Univariable Cox regression analysis of hazard ratios for death in relation to the interaction between DCN‐defined immune cells and tumour cells
**File S20.** Kaplan–Meier analysis and Cox regression of recurrence free survival according to DCN‐defined immune cells interacting with tumour cells

**File S21.** Raw data

## Data Availability

Serum decorin data supporting the findings of this study are found in supplementary material, File S21. Immunofluorescence and immunohistochemical images are provided upon reasonable request.
