# Supplementary material for: Systemic and local decorin levels mirror the clinical course of pancreatic cancer
Source: J Pathol Clin Res. 2025 Dec 14;12(1):e70066. doi: 10.1002/2056-4538.70066 (PMC12702688; doi:10.1002/2056-4538.70066)
Supplement: Supplementary file 1 — File S1. Patient flow chart File S2. Serum sample overview for 124 patients File S3. Detailed methods for multiplexed immunofluorescent images File S4. Kaplan–Meier analyses of overall survival in relation to serum DCN levels at different timepoints in palliative and adjuvant patients File S5. Kaplan–Meier analysis of recurrence free survival in relation to serum DCN levels at baseline in adjuvant patients File S6. Univariable and multivariable Cox regression analyses of death and recurrence in relation to RoC‐D in adjuvant patients File S7. Prognostic value of tertiles of RoC‐D in adjuvant and palliative patients File S8. Number of TMA cores and donor blocks, overall and used in analysis File S9. Compartment‐specific DCN expression according to neoadjuvant treatment File S10. Univariable and multivariable Cox regression analyses of hazard ratios for death and recurrence in relation to DCN expression in the stromal compartment File S11. Prognostic value of conventional clinicopathological parameters in adjuvant patients File S12. Correlations between immune cells and DCN expression in tumour cells and stroma File S13. DCN gene expression in immune cells and immunohistochemical DCN staining in a retrospective cohort File S14. Univariable and multivariable Cox regression analysis of hazard ratios for death and recurrence in relation to DCN positive B cells File S15. Kaplan–Meier analyses of overall and recurrence free survival in relation to different DCN positive immune cell populations File S16. Correlations between DCN expression in different compartments and clinicopathological parameters File S17. Correlations between and DCN expression in tumours and serum DCN levels at baseline File S18. Heatmap of the number of DCN‐defined immune cells interacting with tumour cells File S19. Univariable Cox regression analysis of hazard ratios for death in relation to the interaction between DCN‐defined immune cells and tumour cells File S20. Kaplan–Meier analysis and Cox regr [file CJP2-12-e70066-s002.pdf]

Systemic and local decorin levels mirror the clinical course of pancreatic cancer

M Svensson *et al. J Pathol Clin Res* https://doi.org/10.1002/2056-4538.70066

Supplementary Files S1 – S20
Supplementary File S21 is provided as a separate Excel file.

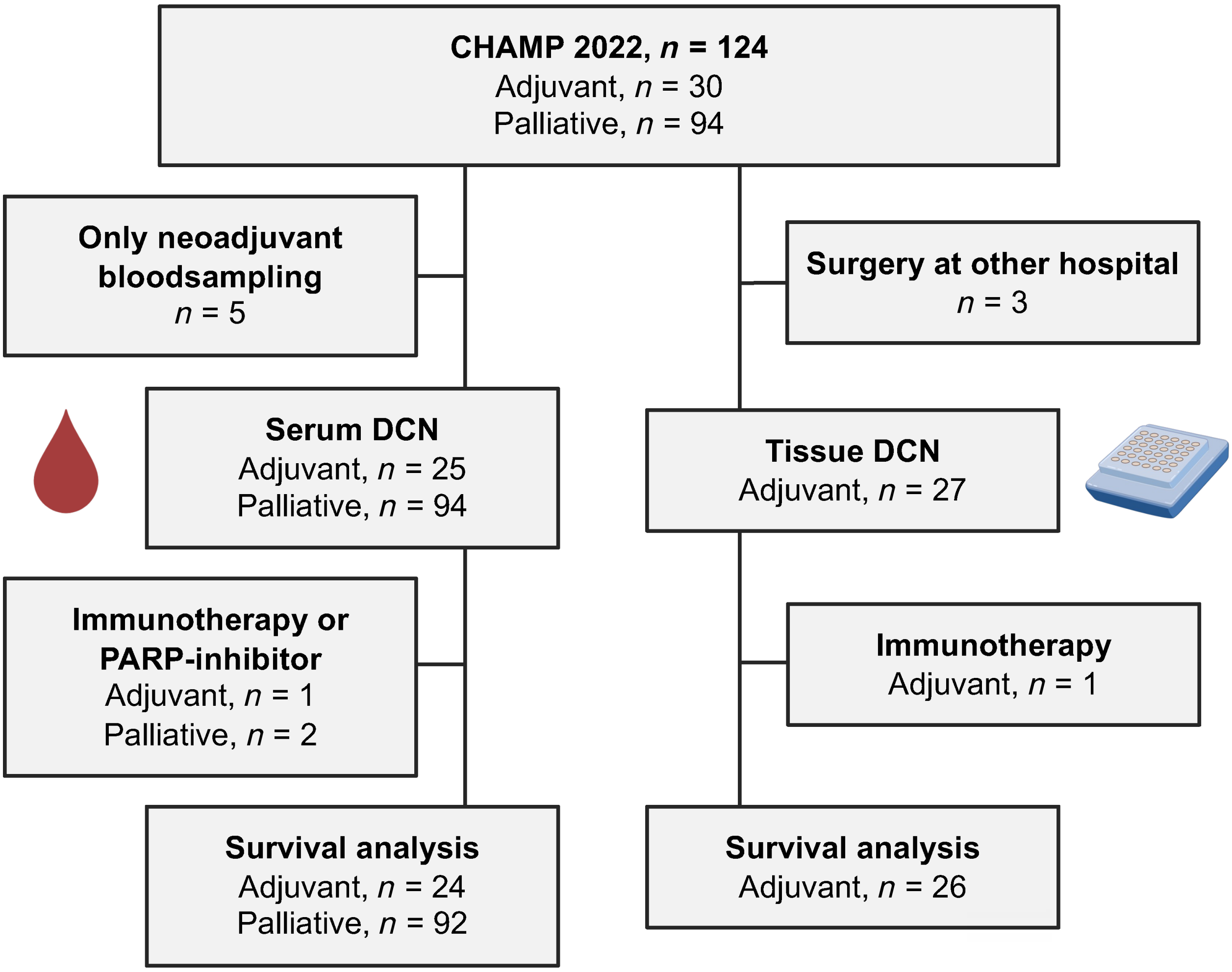

File S1. Flow chart of patient inclusion and exclusion in the different stages of analysis. DCN = decorin.

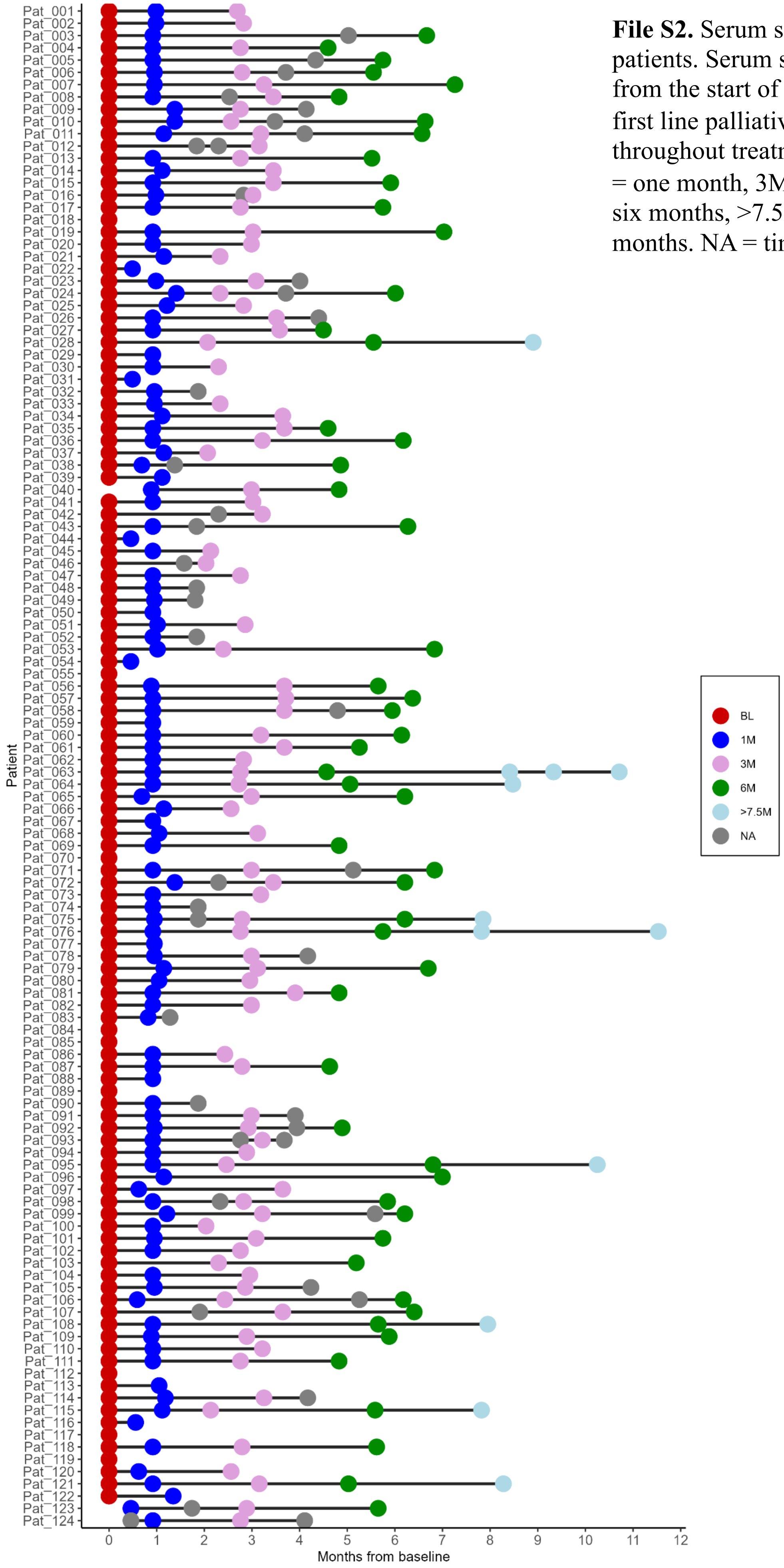

File S2. Serum sample overview for 124 patients. Serum sampling was performed from the start of adjuvant, neoadjuvant or first line palliative chemotherapy and throughout treatment. BL = Baseline, 1M = one month, 3M = three months, 6M = six months, >7.5M = more that 7.5 months. NA = timepoint not assigned.

File S3. Detailed methods for multiplexed immunofluorescent images.

Antibody-fluorophore pairings used in the multiplexed immunofluorescent panel.

Staining order	Antibody	Vendor	Catalogue number	Dilution	Incubation time (min)	Opal pairing*	Dilution	Incubation time (min)
1	CD4	Abcam	AB133616	1:1000	60	Opal 520	1:50	30
2	CD8	Agilent	M710301-2	1:300	30	Opal 570	1:50	30
3	CD20	Agilent	M075501-2	1:1000	30	Opal 480	1:150	10
4	DCN	Sigma Aldrich (Atlas Antibodies)	HPA003315	1:400	30	Opal 620	1:50	10
5	CD68	Agilent	M081401-2	1:300	30	Opal 690	1:50	10
6	PanCK	Agilent	M351501-2	1:300	30	TSA-DIG/Opal 780	1:100/1:25	10 / 60

* Opal kit: NEL861001TK, Akoya Biosciences

Tissue sample preparation

SPTC sections, 4 µm thick, were baked in 60°C overnight, deparaffinized and rehydrated through an alcohol gradient, ending with a 20-minute bath in neutral buffered formalin (Sigma-Aldrich Chemie GmbH) for extra fixation of the tissue.

The antibody-fluorophore pairings, including vendor, dilutions and incubation times are seen in the table below. The sections went through six cycles of manual staining starting with antigen retrieval using a pressure cooker, 2100 Antigen Retriever (Aptum Biologics, Southampton, United Kingdom), followed by washing in tris-buffered saline with Tween, blocking using antibody diluent (Akoya Biosciences, Marlborough, MA) and incubation with the primary antibody. After a washing step, the sections were incubated with secondary horseradish peroxidase (HRP) antibody (Akoya Biosciences) for 10 minutes, followed by washing and staining with an Opal fluorophore (Akoya Biosciences). A final washing step was performed and then the next cycle started. For the sixth cycle, a slightly different fluorophore staining procedure was used. After secondary HRP incubation, the sections were washed and incubated

with tyramide signal amplification-digoxygenin (TSA-DIG) for 10 minutes, followed by an additional antigen retrieval step and finally, staining with Opal 780 fluorophore for one hour. After the sixth and last staining cycle, the sections were counterstained with 4',6-diamino-2 phenylindole (DAPI, Akoya Biosciences) and coverslips were mounted with ProLong Antifade Mountant (Thermo Fischer Scientific, MA).

Scanning

The stained sections were scanned using the PhenoImager HT 2.0 system (Akoya Bioscience) at 20x magnification with onboard unmixing in InForm (version 3.0, Akoya Bioscience) using a synthetic library with a trained autofluorescence library acquired from an unstained section processed in parallel.

Image analysis

Image analysis was performed in QuPath version 0.6.0-rc2 [11]. After omitting cores of bad quality and without cancer cells, tissue objects were created using a pixel thresholder, taking the average intensity of all channels to separate tissue from empty space within each tissue core.

Tumour area (PanCK$^+$), stromal areas and immune rich areas were identified within the tissue objects using a trained pixel classifier. The intensity of decorin (DCN) was measured in the respective area and exported for downstream analysis in R, where the average DCN intensity per area and patient was calculated and used in downstream analysis.

Cell segmentation was performed using InstanSeg [12] within the tissue objects. All channels were used as input to the algorithm, except autofluorescence and DCN, since it is also expressed in the extracellular matrix, to avoid making false cell detections. The cell detections were refined by training object classifiers that detect highly autofluorescent erythrocytes, which were then deleted. To minimize false positive cell classifications in the downstream analysis, cores and areas with high background staining were removed. After this, object classifiers were

trained for each marker, to identify positive cells. For the markers CD4, CD8 and CD20, only cells with a detected nucleus were accepted whereas for CD68 and PanCK, detections without nuclei were accepted as these cells are larger and may have their nucleus in a different plane. The classifiers were then applied one by one, in sequence with the DCN classifier, to all sections and the number of single positive and double positive (marker and DCN) cells per core was exported, along with the measured cell mean intensity of DCN and distance to the nearest PanCK$^+$ cell.

Further analysis was performed in R. The abundance of each cell type was calculated as the number of cells per mm^2 and the mean abundance was calculated per patient and cell type. The fraction of DCN positive cells was defined as the number of DCN positive cells per total cells of that cell type in each core and an average fraction across all cores was calculated per patient. The mean DCN intensity per cell type was calculated for each patient. Similarly, the average DCN intensity of DCN positive cells within each cell type per core was calculated, and finally, the average DCN intensity per patient.

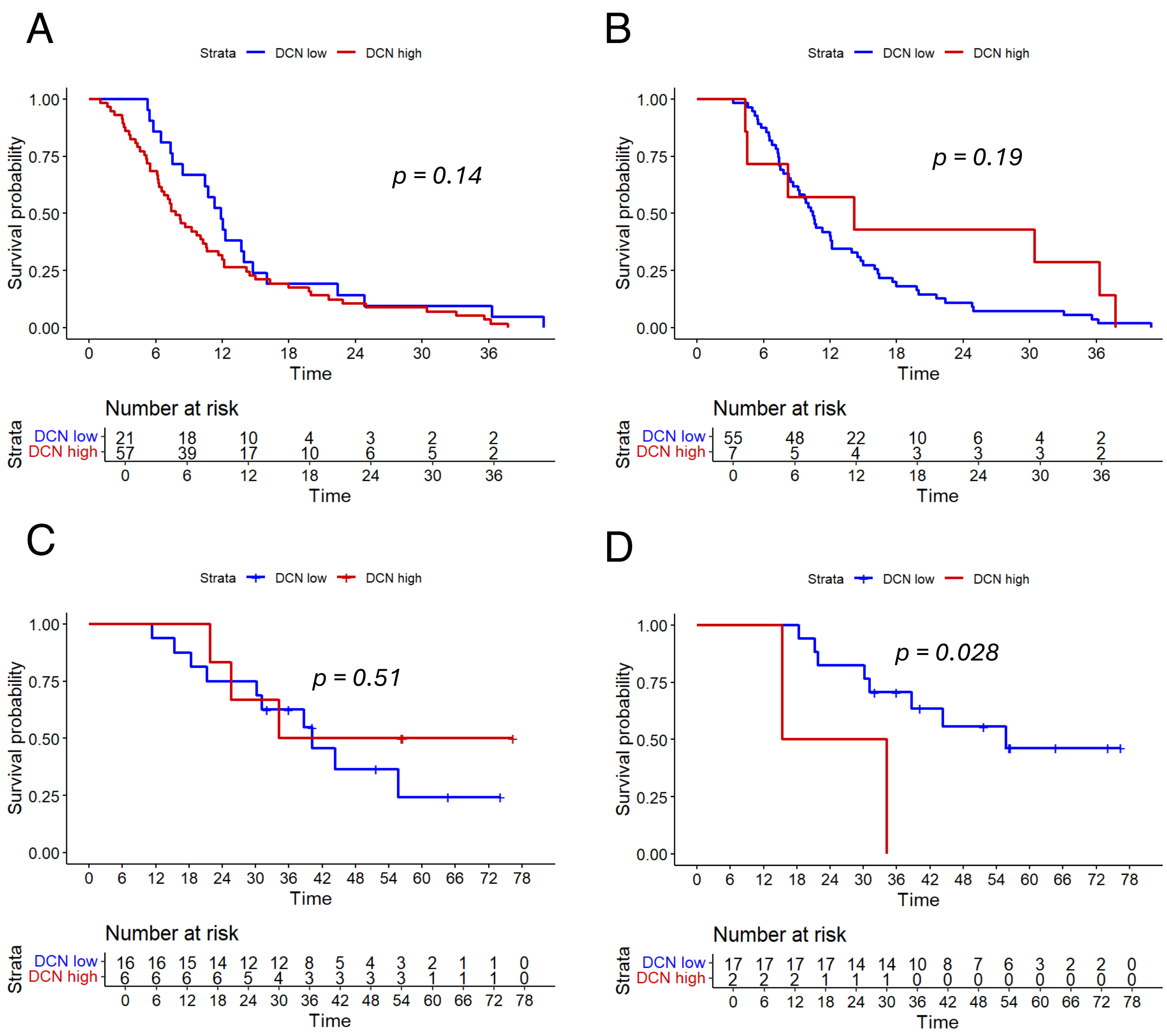

File S4. Kaplan-Meier analysis of overall survival in relation to high and low serum decorin (DCN) levels in palliative patients at (A) one month (cut-off 5.22 NPX) and (B) three months of treatment (cut-off = 5.96 NPX), in adjuvant patients at (C) one month (cut-off 5.39 NPX) and (D) three months of treatment (cut-off 5.62 NPX). The log-rank test was used to calculate the p-values.

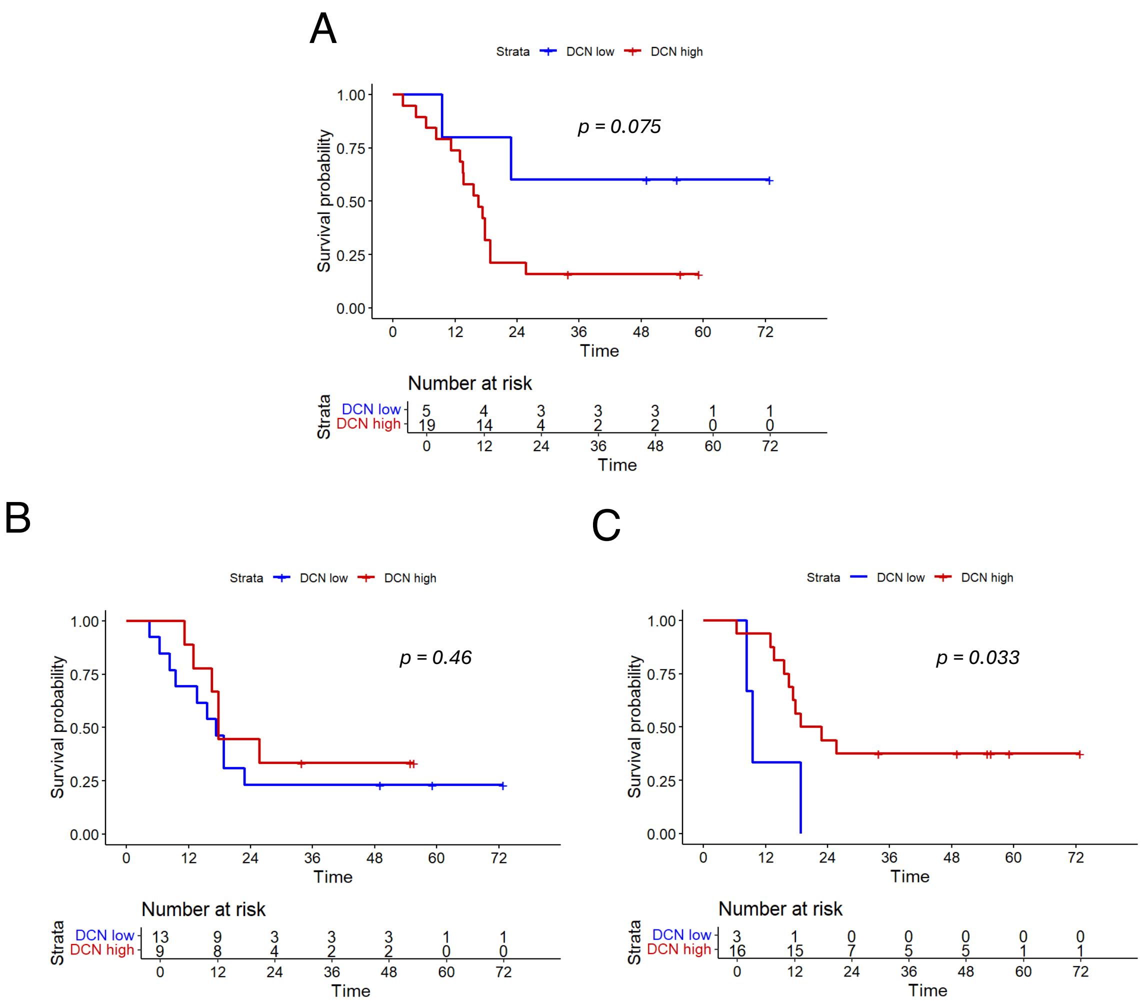

File S5. Kaplan-Meier analysis of recurrence free survival in relation to high and low serum decorin (DCN) levels at (A) BL (cut-off 5.13 NPX), (B) one month (cut-off 5.33 NPX) and (C) three months (cut-off 5.1 NPX) of treatment in adjuvant patients. The log-rank test was used to calculate the p-value.

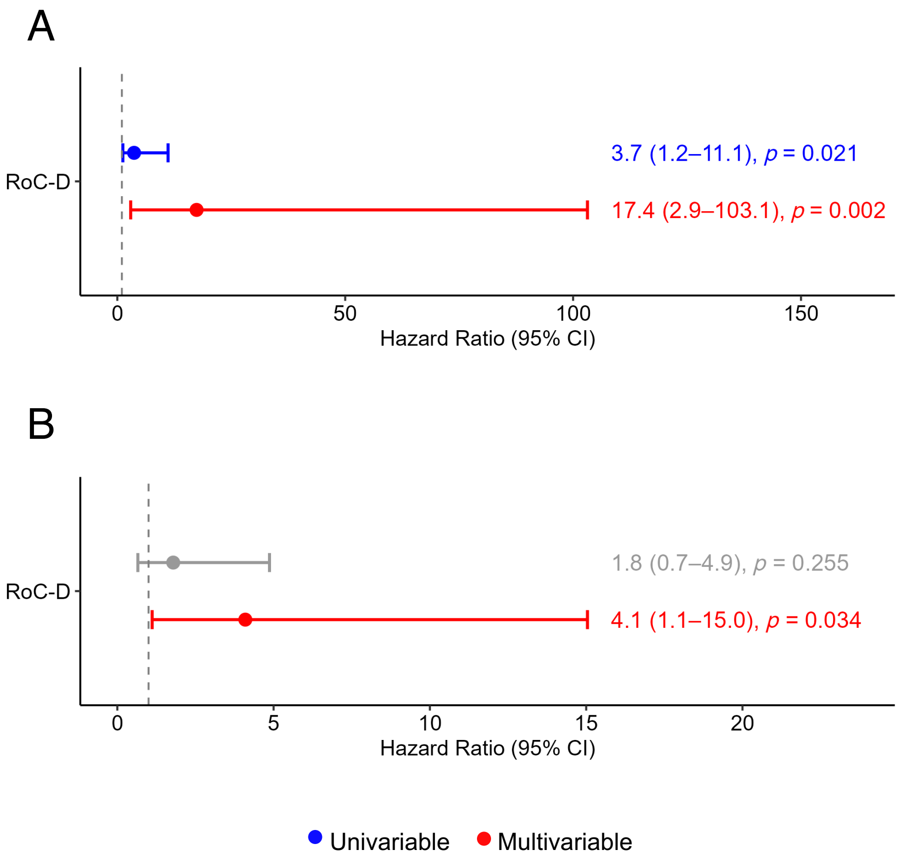

File S6. Univariable and multivariable Cox regression analysis of hazard ratios for (A) death and (B) recurrence in relation to the rate of change of serum DCN (RoC-D) in adjuvant patients (cut-off = 0.15 NPX/month for both OS and RFS). CI = confidence interval.

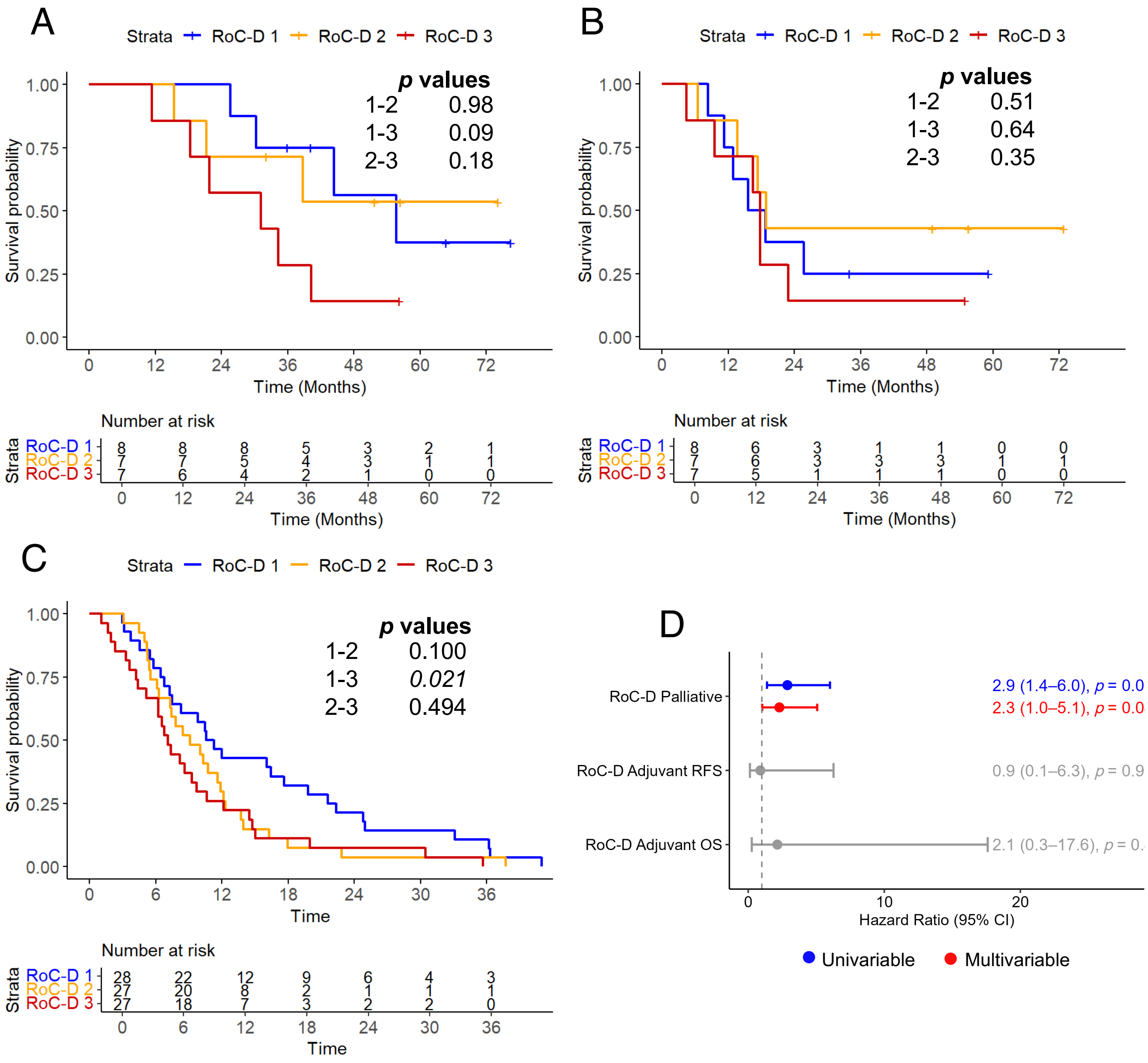

File S7. Kaplan-Meier analysis of (A) overall survival and (B) recurrence free survival in relation to tertiles of the rate of change of DCN (RoC-D) in adjuvant patients, and of (C) overall survival in relation to tertiles of the RoC-D in palliative patients. Log-rank test was applied to calculate the pairwise p-values. (D) Forest plot showing univariable and multivariable hazard ratios for death in palliative patients and univariable hazard ratios for death and recurrence in adjuvant patients according to RoC-D in continuous units. The multivariable analysis was adjusted for performance status, regimen and metastatic disease. CI = Confidence interval, RFS = recurrence free survival, OS = overall survival.

File S8. Total number of cores and donor blocks used in the SPTC construction, and number of 1 mm TMA cores used in the multiplex immunofluorescent analysis, per patient. Damaged cores and cores with high autofluorescence were excluded and only cores containing tumour cells were used in the analyses, excluding areas of normal pancreatic tissue.

Patient	Total cores	Number of blocks	Cores per block	Cores used in analysis
Pat_011	55	17	3.2	19
Pat_013	45	17	2.6	10
Pat_015	50	19	2.6	18
Pat_023	47	16	2.9	14
Pat_029	21	11	1.9	11
Pat_033	53	8	6.6	27
Pat_035	84	14	6.0	41
Pat_037	42	18	2.3	15
Pat_038	98	17	5.8	60
Pat_045	80	16	5.0	29
Pat_047	60	10	6.0	13
Pat_048	82	14	5.9	50
Pat_056	30	7	4.3	17
Pat_057	68	8	8.5	33
Pat_058	48	8	6.0	32
Pat_061	39	14	2.8	4
Pat_063	78	23	3.4	29
Pat_069	42	8	5.3	8
Pat_071	105	16	6.6	68
Pat_079	48	8	6.0	4
Pat_085	84	11	7.6	31
Pat_087	120	24	5.0	51
Pat_096	39	6	6.5	19
Pat_104	63	9	7.0	33
Pat_109	55	11	5.0	20
Pat_111	51	5	10.2	17
Pat_118	67	6	11.2	33

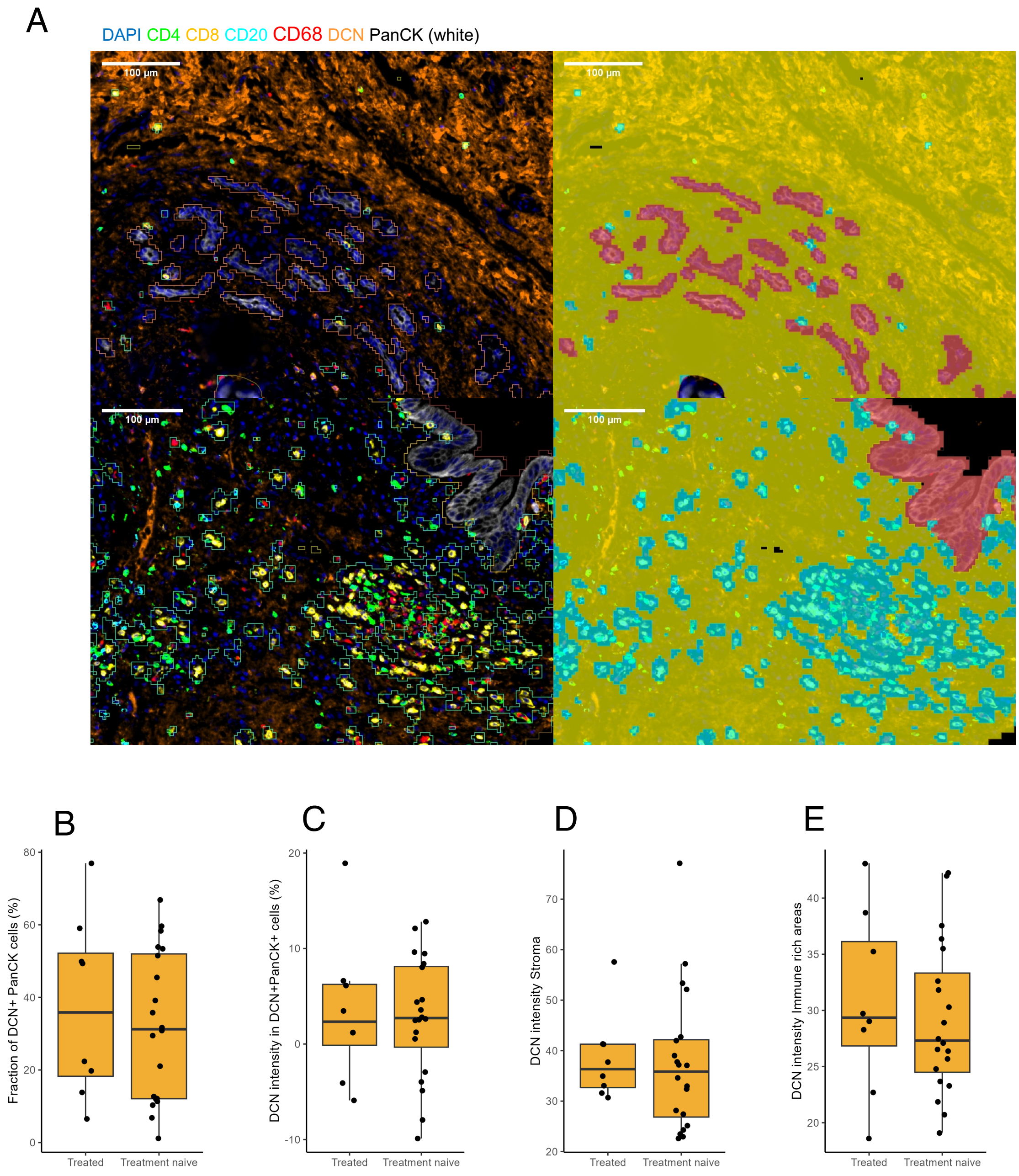

File S9. (A) Representative multiplexed immunofluorescent images of tumour (red), stroma (yellow) and immune rich (cyan) areas in two cases representing high (top) and low (bottom) stromal decorin (DCN). (B) The fraction and (C) intensity of DCN expression in PanCK$^+$ tumour cells, the intensity of DCN in (D) stroma and in (E) immune rich areas in treatment naive and treated tumours, respectively.

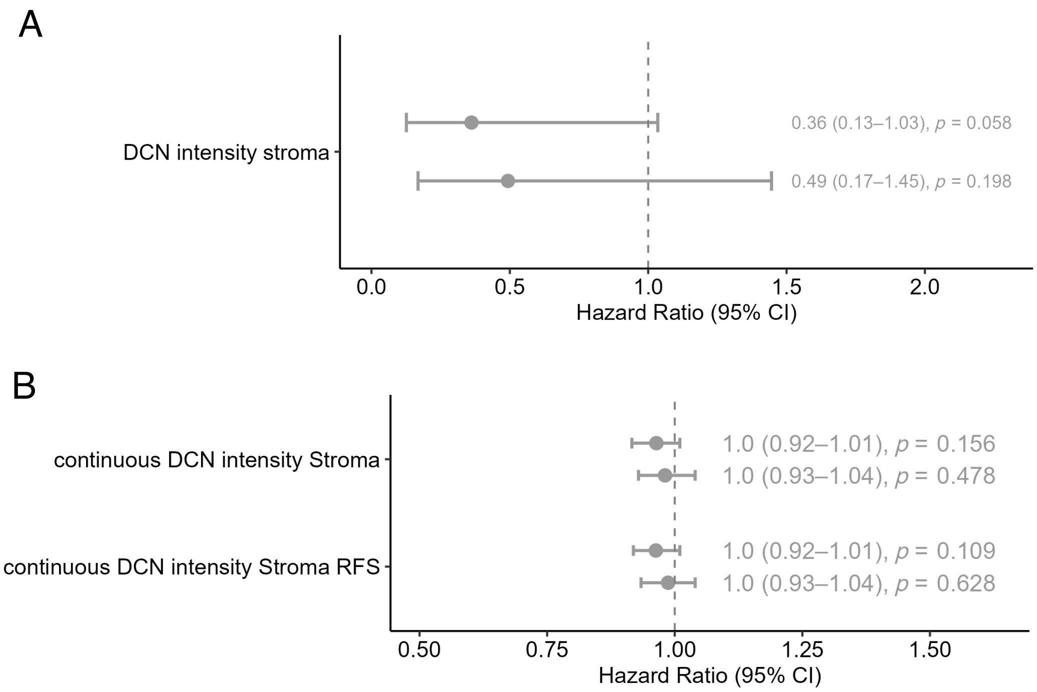

File S10. (A) Univariable and multivariable Cox regression analysis of hazard ratios for death in relation to the intensity of stromal decorin (DCN) expression dichotomized according to high and low DCN intensity in stroma (cut-off 32.39 AU). (B) Univariable Cox regression analysis of hazard ratios for death and recurrence, respectively, in relation to the intensity of stromal DCN expression, in continuous units. CI = confidence interval.

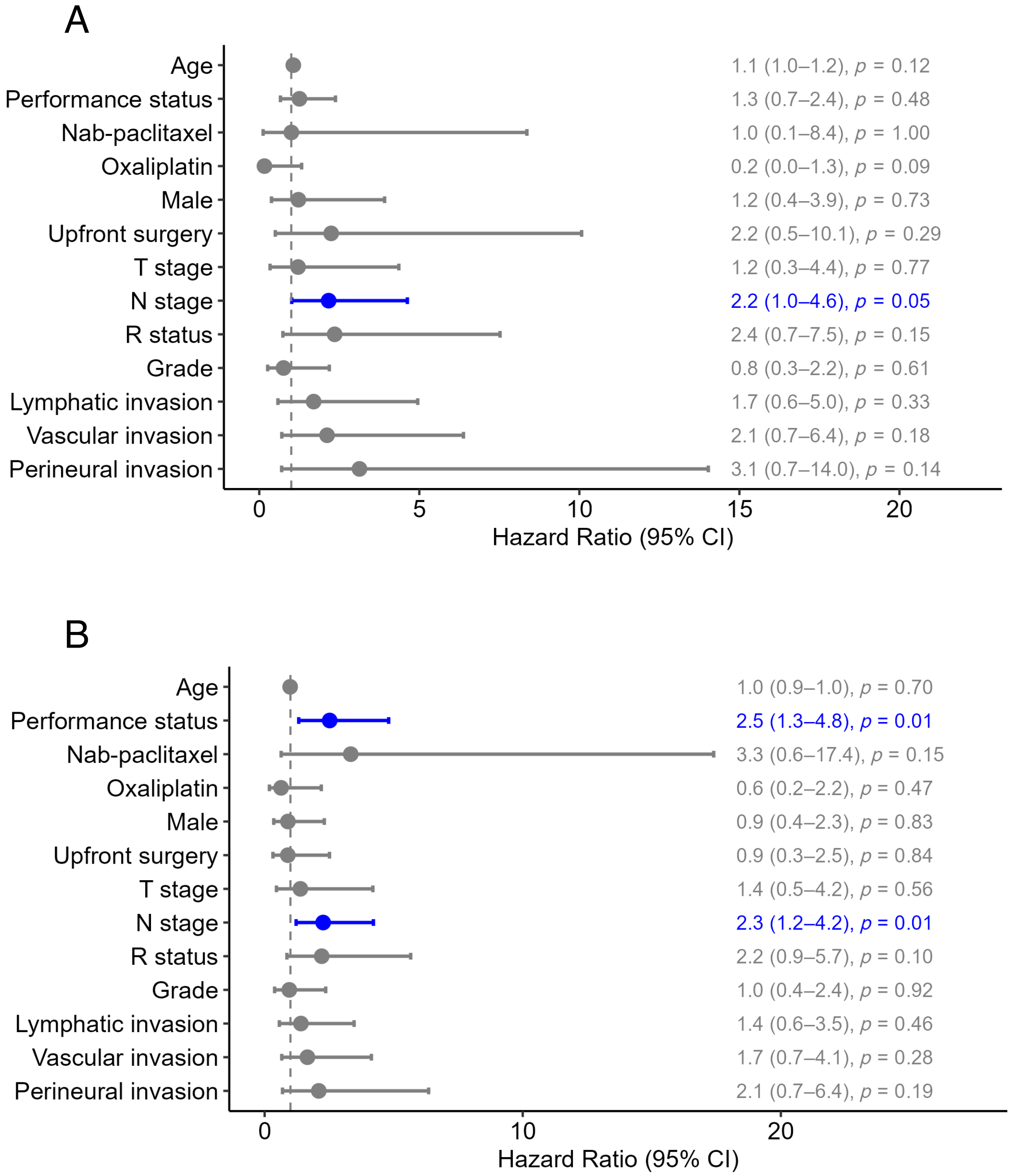

File S11. Univariable Cox regression analysis of hazard ratios for (A) death and (B) recurrence in relation to conventional clinicopathological parameters in adjuvant patients (n = 24). Gemcitabine is the reference for Nab-paclitaxel and oxaliplatin. R status = negative *versus* positive resection margins, T stage = T1-T2 *versus* T3-T4, N-stage = N0, N1, N2, R status = negative *versus* positive resection margins, grade = well and moderately *versus* poorly differentiated. CI= confidence interval.

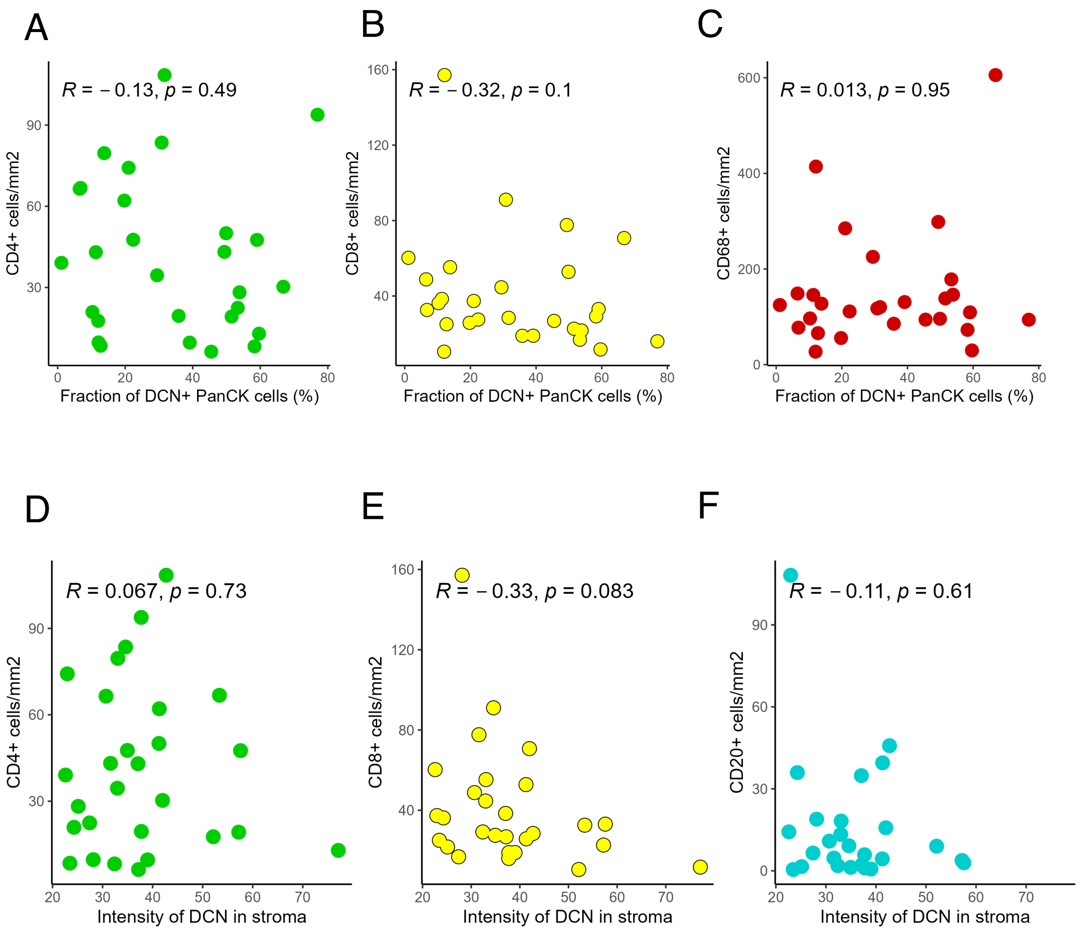

File S12. Spearman's correlation between the number of (A) $CD4^{+}$ T helper cells, (B) $CD8^{+}$ cytotoxic T cells and (C) $CD68^{+}$ macrophages and the fraction of decorin (DCN) in $PanCK^{+}$ tumour cells, and between the number of (D) $CD4^{+}$ T helper cells, (E) $CD8^{+}$ cytotoxic T cells, and (F) $CD20^{+}$ B cells and stromal decorin intensity.

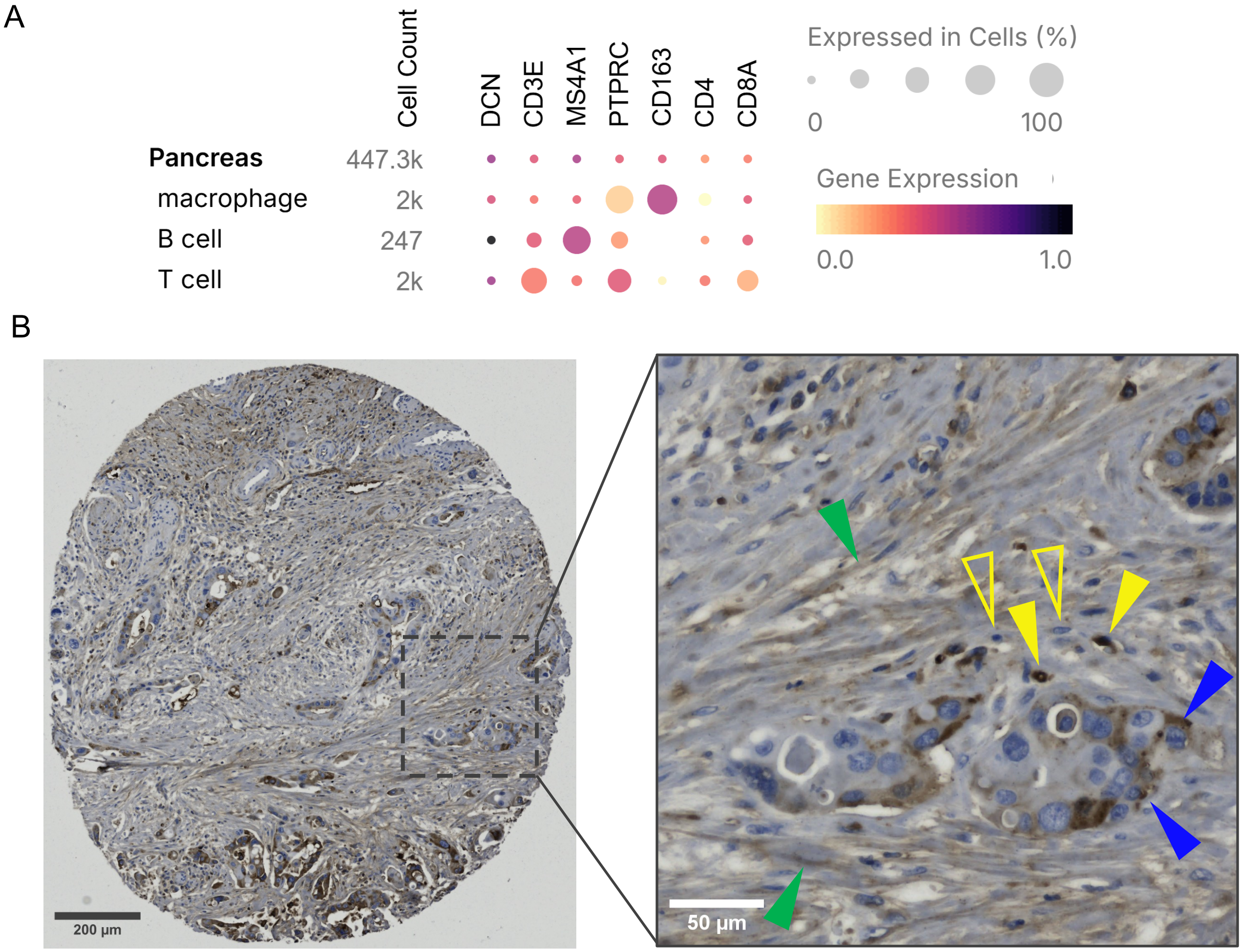

File S13. Decorin (DCN) gene expression was investigated in T cells, B cells and macrophages from human pancreas using three publicly available datasets in the CZ CELLxGENE Discover tool for validatory purposes (A). Sample image of single immunohistochemical (IHC) DCN staining in PDAC samples from a retrospective cohort (B). Four µm thick sections, were pretreated for antigen-retrieval in the automated PT-link module (Dako, Glostrup, Denmark). IHC staining with anti-DCN (dilution 1:250, HPA003315, Sigma Aldrich, Atlas Antibodies) was performed using Autostainer Link 48 and EnVision FLEX+ Detection System (Dako) and counterstained with hematoxylin. Green arrowheads = DCN positive collagen fibres, filled yellow arrowheads = DCN positive immune cell, hollow yellow arrowhead = DCN negative immune cell, blue arrowhead = DCN positive tumour cell.

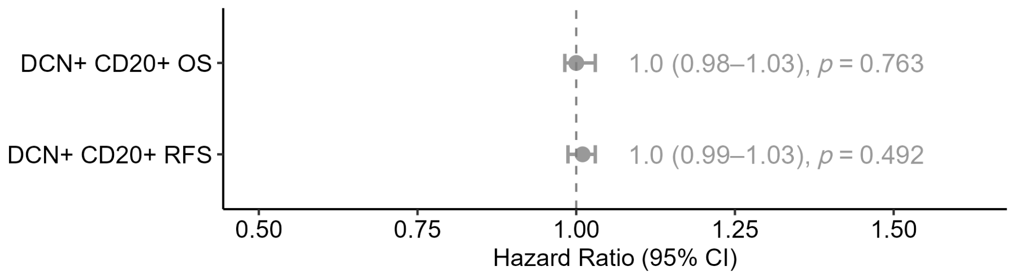

File S14. Forest plot showing univariable hazard ratios for death (OS) and recurrence (RFS) in adjuvant patients (n=25) according to the fraction of $DCN^{+}CD20^{+}$ B cells in continuous units. DCN= decorin, CI = confidence interval.

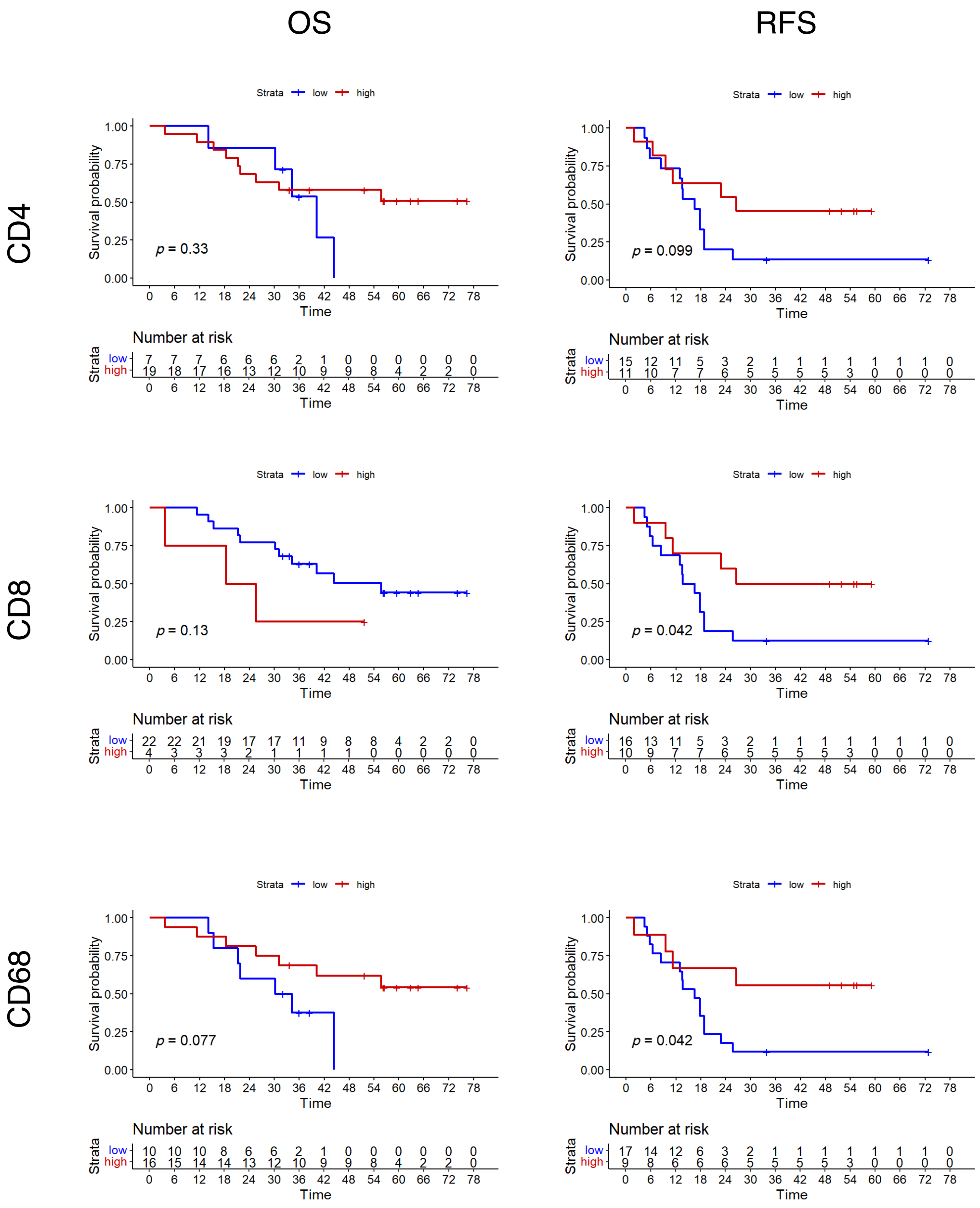

File S15. Kaplan-Meier analysis of overall survival (OS) and recurrence free survival (RFS) in relation to high and low expression of different decorin (DCN) positive immune cell populations. The division into high and low was obtained by applying the optimal prognostic cut-off for each immune subset. CD4^{+}DCN^{+} cut-off for OS: 10.9%, cut-off for RFS: 37.4%. CD8^{+}DCN^{+} cut-off for OS: 71.2%, cut-off for RFS: 48.8%. CD68^{+}DCN^{+} cut-off for OS: 23.0%, cut-off for RFS: 43.9%.

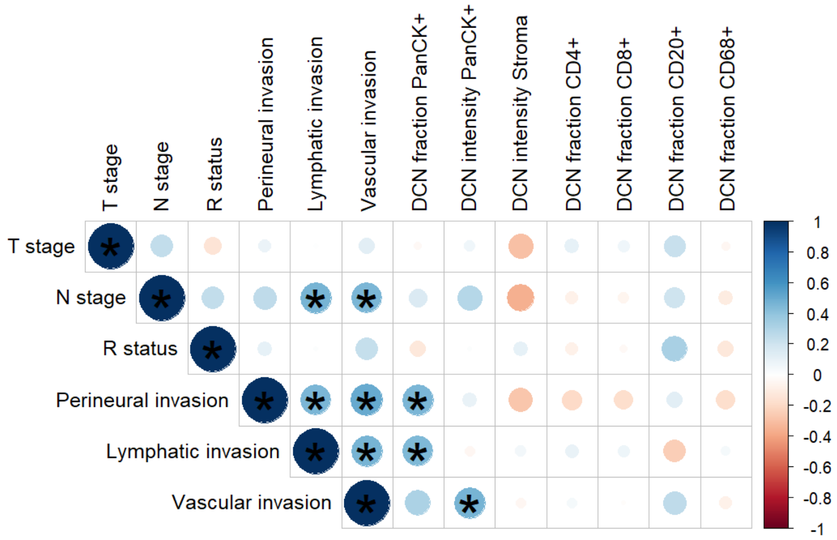

File S16. Spearman's correlation coefficients between clinicopathological parameters and the fraction or intensity of decorin (DCN) expression in tumour cells, stroma and immune cells.

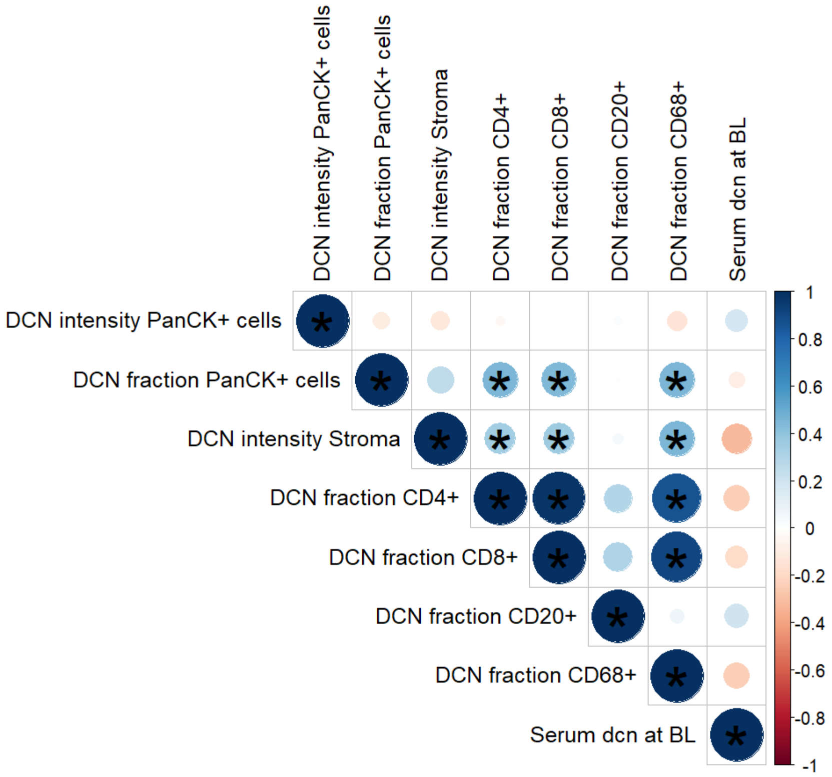

File S17. Spearman's correlation coefficients between decorin (DCN) expression in tumour cells, stroma and different immune cell populations and serum decorin levels at baseline in adjuvant patients. BL = baseline.

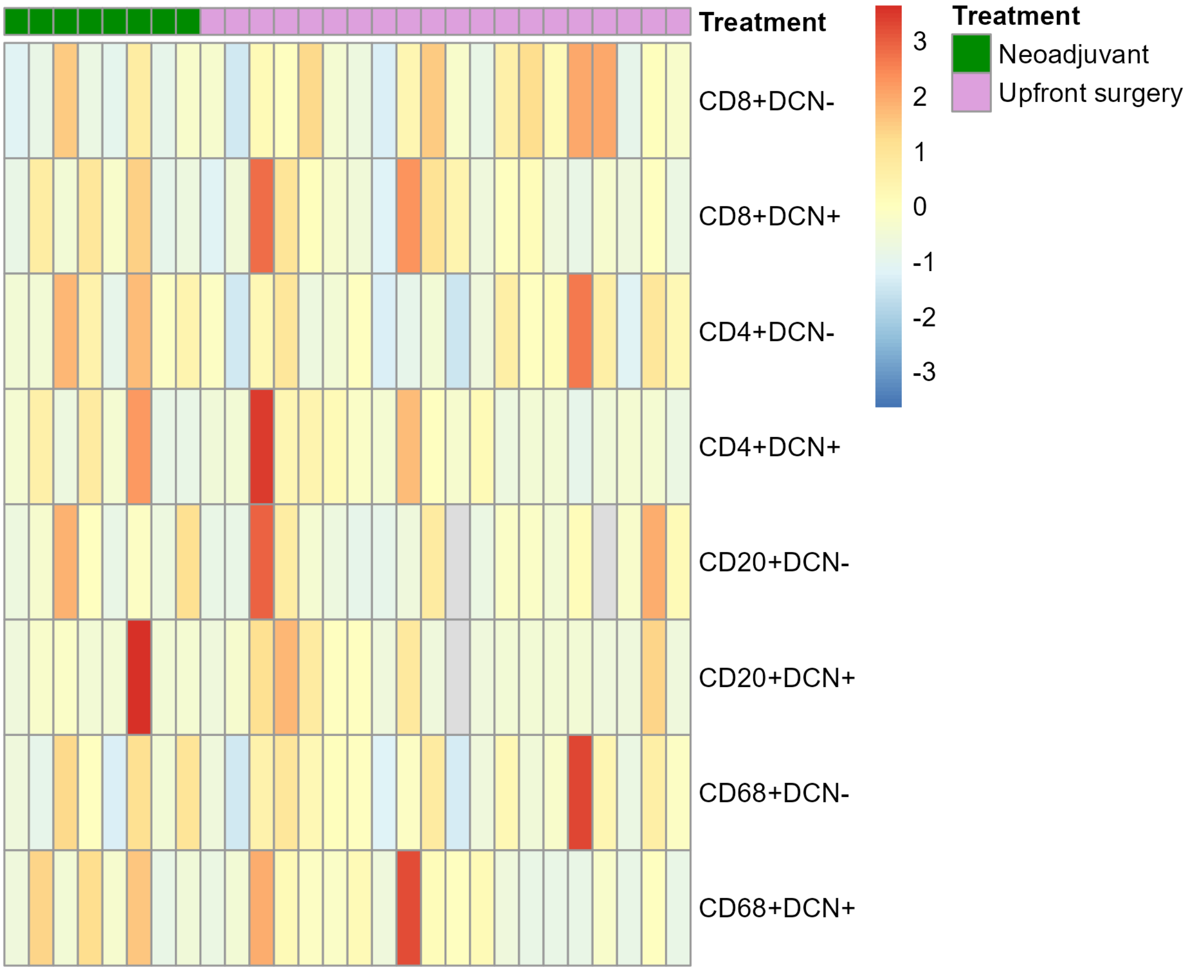

File S18. Heatmap of the mean number of decorin-defined immune cells interacting with tumour cells per total number of immune cells of the specific subset per patient, normalised using Z-score. DCN = decorin.

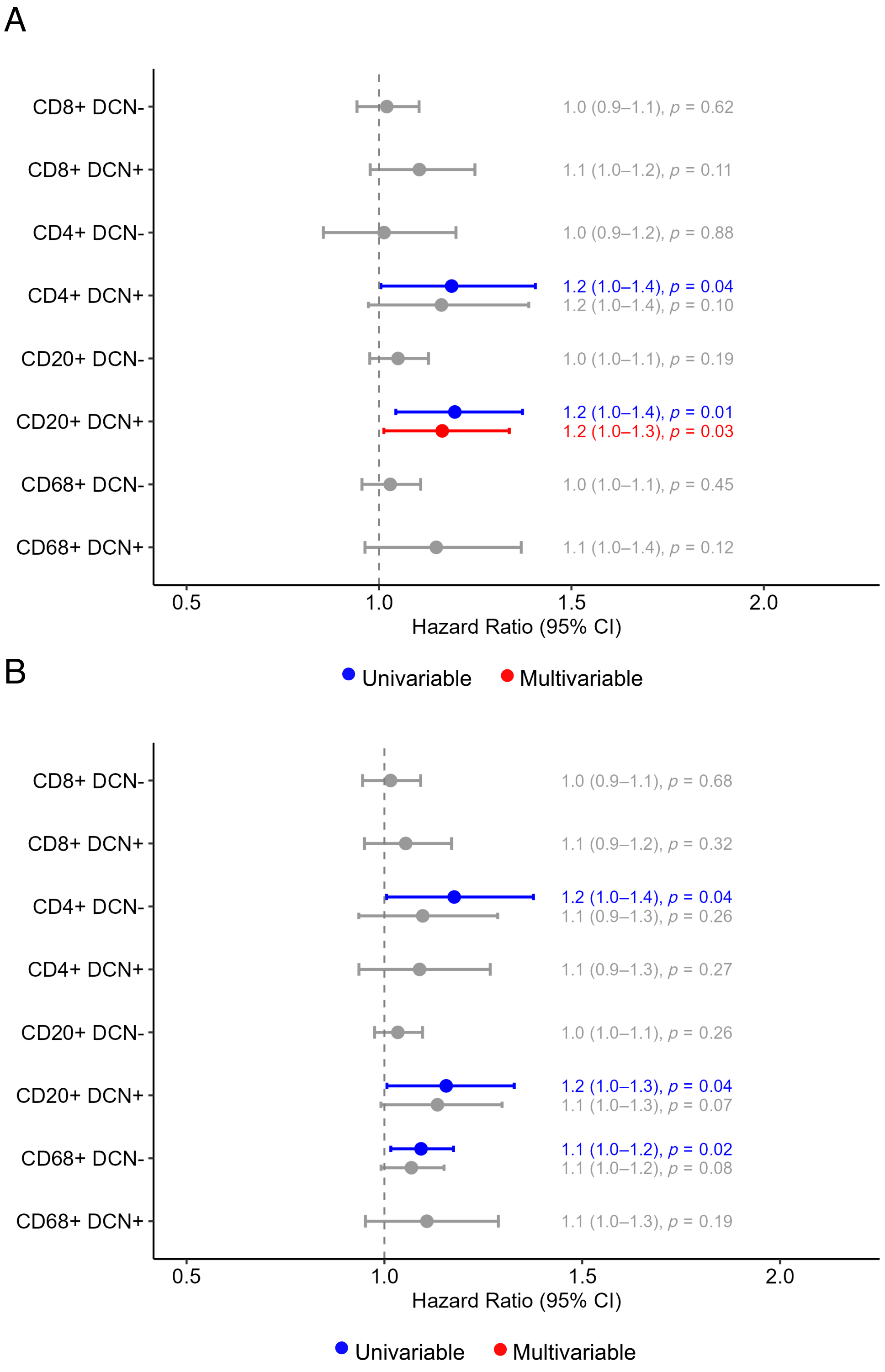

File S19. Univariable and multivariable Cox regression analysis of hazard ratios for (A) death and (B) recurrence in relation to the fraction of decorin-defined immune cell subsets interacting with tumour cells. DCN = decorin, CI = confidence interval.

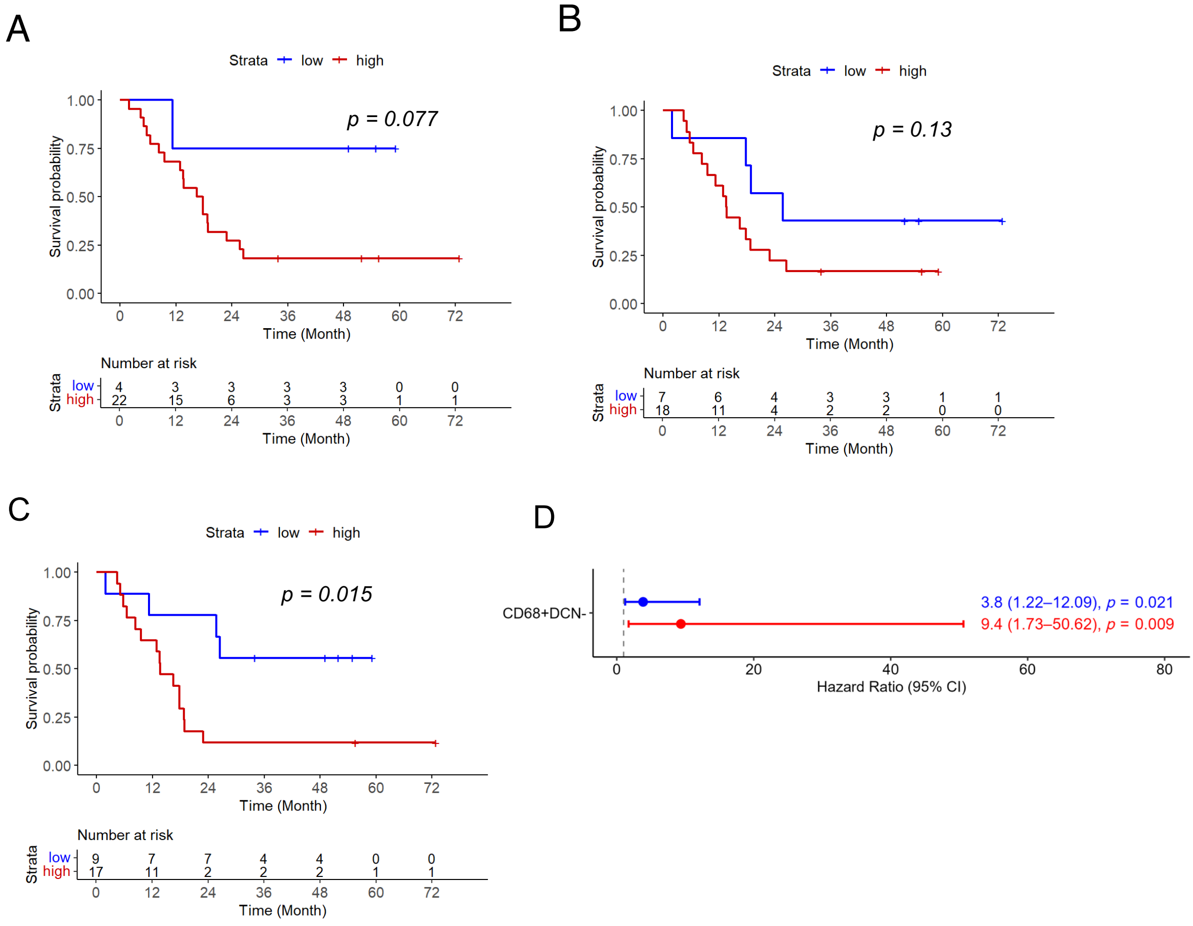

File S20. Kaplan-Meier analysis of recurrence free survival according to high and low fractions of (A) CD4^{+}DCN^{-} T cells (cut-off: 1.82%) and (B) CD20^{+}DCN^{+} B cells (cut-off: 0.11%) and (C) CD68^{+}DCN^{-} macrophages (cut-off: 6.86%) interacting with tumour cells. Log-rank tests were applied to calculate p-values. (D) Univariable and multivariable Cox hazard ratios for recurrence according to high and low fraction of CD68^{+}DCN^{-} macrophages interacting with tumour cells (same cut-off as C). CI = confidence interval, DCN = decorin. The multivariable analysis was adjusted for N stage and performance status.
